# Genetic Control of Reproductive Traits under Different Temperature Regimes in Inbred Line Populations Derived from Crosses between *S. pimpinellifolium* and *S. lycopersicum* Accessions

**DOI:** 10.3390/plants11081069

**Published:** 2022-04-14

**Authors:** Maria Jose Gonzalo, Luciano Carlos da Maia, Inmaculada Nájera, Carlos Baixauli, Giovanni Giuliano, Paola Ferrante, Antonio Granell, Maria Jose Asins, Antonio Jose Monforte

**Affiliations:** 1 Instituto de Biología Molecular y Celular de Plantas, Universitat Politècnica de València-Consejo Superior de Investigaciones Científicas, 46010 Valencia, Spain; magonpa1@upvnet.upv.es (M.J.G.); lucianoc.maia@gmail.com (L.C.d.M.); agranell@ibmcp.upv.es (A.G.); 2 Plant Genomics and Breeding Center, Faculdade de Agronomia Eliseu Maciel, Universidade Federal de Pelotas, Pelotas 96010-610, RS, Brazil; 3 Centro de Experiencias de Cajamar en Paiporta, 46200 Paiporta, Spain; inmaculadanajera@fundacioncajamar.com (I.N.); carlosbaixauli@fundacioncajamar.com (C.B.); 4 Agenzia Nazionale Per Le Nuove Tecnologie, L’energia e Lo Sviluppo Economico Sostenibile (ENEA), Casaccia Research Centre, Via Anguillarese 301, 00123 Rome, Italy; giovanni.giuliano@enea.it (G.G.); paola.ferrante@enea.it (P.F.); 5 Instituto Valenciano de Investigaciones Agrarias (IVIA), Carretera Moncada-Náquera, Km 4.5, Moncada, 46113 Valencia, Spain; mjasins@ivia.es

**Keywords:** abiotic stress, heat tolerance, fruit set, QTL, epistasis

## Abstract

In the present work, we study the genetic control of reproductive traits under different heat stress conditions in two populations of inbred lines derived from crosses between two *S. pimpinellifolium* accessions and two tomato cultivars (E9×L5 and E6203×LA1589). The temperature increase affected the reproductive traits, especially at extremely high temperatures, where only a few lines were able to set fruits. Even though a relative modest number of QTLs was identified, two clusters of QTLs involved in the responses of reproductive traits to heat stress were detected in both populations on chromosomes 1 and 2. Interestingly, several epistatic interactions were detected in the E9×L5 population, which were classified into three classes based on the allelic interaction: dominant (one locus suppressed the allelic effects of a second locus), co-adaptive (the double-homozygous alleles from the same parent alleles showed a higher phenotypic value than the combination of homozygous alleles from alternative parents) and transgressive (the combination of double-homozygous alleles from different parents showed better performance than double-homozygous alleles from the same parents). These results reinforce the important role of non-additive genetic variance in the response to heat stress and the potential of the new allelic combinations that arise after wide crosses.

## 1. Introduction

The Intergovernmental Panel on Climate Change (IPCC) estimates an increase in average temperatures from 2 to 4.5 °C within the 21st century [1]. In this scenario of global warming, agriculture is expected to be one of the most affected sectors [2], with negative impacts on crop productivity due to the effects of high temperatures on plant development [3,4]. The major losses due to heat stress are expected to occur in low-latitude regions (temperate and tropical areas), with temperatures exceeding even the most extreme seasonal temperatures recorded to date [5]. In fact, yield reductions due to heat stress have already been documented in many crops from these geographical areas, such as wheat, rice, barley, sorghum, maize, chickpea, canola and more [6,7,8].

Tomato (*Solanum lycopersicum* L.) is one of the most extensively cultivated crops in temperate regions worldwide [9]. The cultivation of this crop is affected by high temperature, which can cause different physiological or morphological injuries, such as reductions in plant growth and development or damage to reproductive organs and pollen [10,11,12,13]. As a consequence, agronomic yield reductions have already been reported due to heat stress [6].

Thus, there is an urgent need to increase the knowledge of the genetic responses to heat stress, considering that heat tolerance is a complex trait affected by numerous factors [14], and to develop plant breeding strategies to face this problem. Reproductive traits have been commonly used to study the responses to heat stress in tomato plants. The flower number per inflorescence decreases due to heat stress [15,16], and the ability of plants to set fruit after the exposure to high temperatures is the most commonly studied trait. This trait is quite complex, involving physiology, biochemistry and gene regulation pathways [3], affecting aspects ranging from the pollen viability [12,14,17], photosynthesis and respiration [18] to activation or silencing of genes [19,20]. Additionally, the fruit set is directly correlated with the final agronomic yield. Therefore, the fruit set at high temperature is considered a good indicator of heat tolerance in tomatoes, and it has been widely studied in previous works [14,16,21,22,23]. Only a very limited number of cultivated varieties or accessions have been identified as heat-tolerant [10,23,24]. On the other hand, several wild *Solanum* spp. are well adapted to marginal environments, being more tolerant to biotic and abiotic stresses than cultivated varieties [25], which could be potential sources of tolerance. In tomatoes, the use of these wild relative species has been extensively exploited in plant breeding strategies for different characteristics [26], including biotic stress [25] or abiotic stress, such as salinity tolerance [27,28,29]. Regarding heat tolerance, accessions from wild *Solanum* spp. such as *S. pimpinellifolium*, L., *S. pennellii* L., *S. habrochaites* L. and *S. chmielewskii* L. have been found to be tolerant to high temperatures [14,17,23,30,31,32,33,34], confirming the value of the wild germplasm as a source of thermotolerance.

Currently, our understanding of the genetic control of the heat tolerance in tomatoes is still very limited, with few reports addressing this topic. Quantitative trait loci (QTLs) involved in heat tolerance have been previously identified in tomatoes [22,24,35,36,37,38,39]. In most of these studies, the identification of QTLs associated with heat tolerant traits in tomatoes was achieved using simple mapping populations (F_2_), with a limited sample size or without a dense marker coverage. Therefore, the number of reported QTLs involved in heat tolerance is relatively low, and the stability of their effects still needs to be verified. More recently, powerful mapping populations such as recombinant inbred lines (RILs), introgression lines (ILs) [22], multiparent advanced generation intercross (MAGIC) panels and genome-wide association study (GWAS) panels [39] have been used to dissect the genetic control of heat tolerance, providing the foundation for marker-assisted selection programs for the development of tolerant cultivars. 

Along with the temperature, other environmental factors such as salinity and drought affect plant growth [40]. Furthermore, in real field conditions, the occurrence of different stresses is common. The response to the combination of stresses is specific and independent of the individual response to each stress [41], although cross-tolerance can occur [42]. For example, the effect of heat stress has been reported to increase tolerance to various abiotic stresses [43]. In the current climate change scenario, the concurrence of various stresses simultaneously is expected. Thus, the search for plants with tolerance to different stresses will be a target in plant breeding programs. In the same way, increased knowledge of the genetic control of tolerance to high temperature has become necessary, together with the development of new varieties of tomato tolerant to heat and to different abiotic stresses. Therefore, taking these perspectives into account, the main objective of the present work is the identification of QTLs that are mainly related to reproductive traits involved in the responses to different temperatures. Two very different populations of inbred lines from independent crosses between tomato accessions (fresh market and processing tomato cultivars) and two *S. pimpinellifolium* accessions were studied to expand the genetic variability, and to genetically analyze the responses of reproductive traits to heat stress. 

## 2. Results

### 2.1. E9×L5 Population

#### 2.1.1. Phenotypic Variations in Reproductive Traits at Different Temperatures

**Figure 1:**
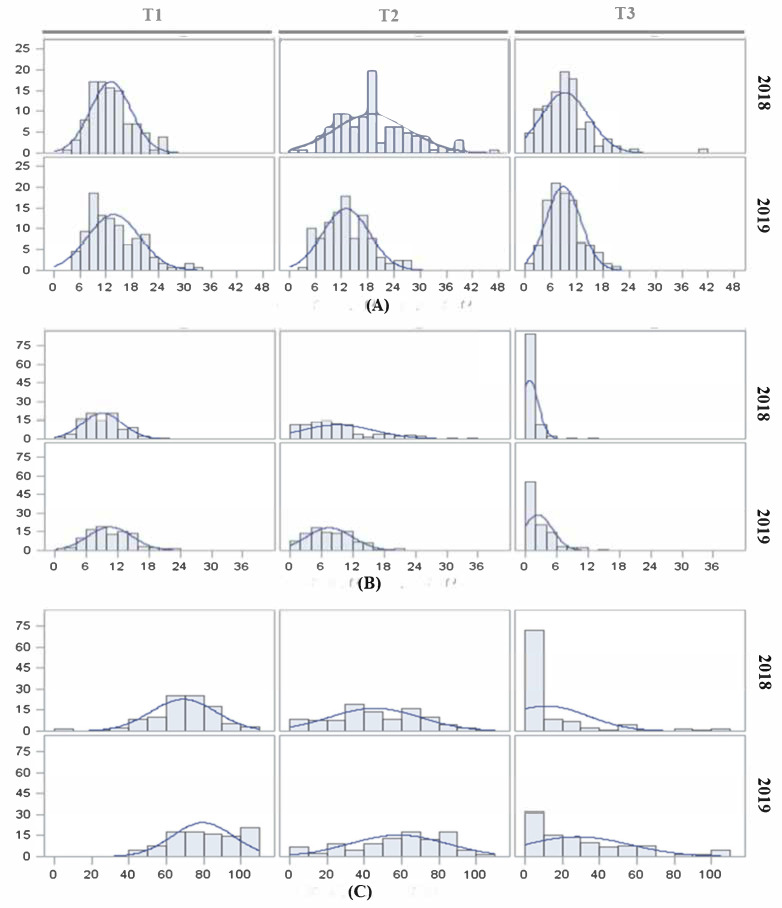
Histograms depicting the distribution of reproductive traits in the RIL population E9×L5. Reproductive traits, (**A**) flower numbers (FLN), (**B**) fruit numbers (FRN) and (**C**) fruit sets (FRS) were studied in 2018 and 2019 under three temperature regimes (T1: 25 °C day/20 °C night; T2: 30 °C day/25 °C night; T3: 35 °C day/30 °C night).

Trait distributions for each temperature regime and year are represented in Figure 1. Means, standard deviations and ranges are also depicted in Supplementary Table S1. FLN means and distributions were similar between T1 and T2, with a relatively modest mean decrease under T3 (Figure 1A). In the case of FRN, a more severe reduction in fruit number concomitant with the temperature increase was observed, with the distribution dramatically skewed to low values under T3 (Figure 1B). Similarly, the FRS distribution was skewed to low values under T3 (Figure 1C).

Therefore, the FRN and particularly FRS were the traits most affected by the temperature increases, especially under T3. It is noteworthy that FRS showed a wide range, with several RILs capable of setting fruit, even at extremely high temperatures, suggesting the presence of a genetic variability for setting fruit under heat stress in this population. 

Additionally, to confirm the consistency of the results, the correlations within and between years in the three temperature regimes were analyzed for all reproductive traits. Correlations within years and between years for FLN were highly significant and positive in every temperature regime (Table 1A). For the FRN values, the correlations between years were positive and highly significant under T1 and T2 regimes, and lower but still significant under T3. Correlations within years were highly significant in 2019, but in 2018 the only significant correlation was observed between T1 and T2 (Table 1B). The correlations between years for FRS were not significant under T1 and T2, whereas a low and positive correlation was observed under T3 for this population. Moreover, FRS correlations between temperature regimes were only significant in 2019 (Table 1C).

Moreover, the repeatability of each trait was calculated as an estimator of broad-sense heritability [44]. This analysis of repeatability showed higher repeatability for the optimal temperature T1, but it decreased as the temperature increased. The coefficient of repeatability for both non-transformed and log-transformed data also suggested that the traits with the highest broad-sense heritability were FLN and FRN under T1 and T2. The FLN also presented a high repeatability under T3 (Table 2). On the other hand, plasticity indexes showed low repeatability, indicating low heritability for these traits. 

#### 2.1.2. Identification of QTL in the RIL Population E9×L5

The multi-environmental QTL analysis results are shown in Table 3, while Supplementary Table S2 contains the QTLs detected separately each year. The multi-environment QTL analysis was also divided into the three categories (non-transformed data, log-transformed data and plasticity indexes). Thus, a total of 31 QTLs involved in the reproductive traits at different temperatures were identified (15 for non-transformed data, 11 for log-transformed data and 5 for plasticity indexes) (Table 3).

Among the 15 QTLs identified using the non-transformed data, six were detected for FLN (three under T1 and three under T2), with the L5 allele increased the flower number. Nine QTLs were detected for FRN, seven of them under T1, distributed in five linkage groups, with two of them under T2. These two QTLs under T2 co-localized with QTLs identified for FLN and FRN under T1. FRN QTL *frn1.1_T2_2E*, localized in chromosome 1 at around 48 cM with the L5 allele, increasing the fruit number and co-segregated with QTLS involved in FLN and FRN under T1, with the last ones being the QTLs with the highest effects (R^2^ > 20%). Moreover, *frn2.1_T2_2E* also co-segregated with the FLN QTL *fln2.1_T2_2E* (Table 3; Supplementary Figure S1). No significant QTLs were detected under T3 nor at any other temperature for FRS. 

Next, the QTL analysis was implemented with log-transformed data to mitigate the effects of the non-normal distribution on the QTL mapping. A total of eleven QTLs were identified: five for LogFLN, four for LogFRN and two for LogFRS (Table 3; Supplementary Figure S1); six of them were not detected with the non-transformed data. The QTLs with the higher effects were detected in chromosomes 1 and 2 (*flnlog1.1_T1_2E*, *flnlog2.1_T2_2E*, R^2^ > 15%). Interestingly, the same region on chromosome 2 harbored QTLs for LogFLN and LogFRN at different temperature regimes, along with L5 allele increasing the traits. Moreover, the log transformation allowed the identification of QTLs at extremely high temperature: *flnlog6.1_T3_2E* with L5 increasing the trait and R^2^ = 7.16. Finally, two QTLs in chromosome 5 were detected for LogFRS: *frslog5.1_T3_2E* at 16 cM, with R^2^ = 8.47 and the E9 allele increasing the trait, and *frslog5.2_T3_2E* at 55 cM, with R^2^ = 6 and the L5 allele increasing the trait (Table 3). 

Five QTLs were found for plasticity traits. Four of them corresponded to plasticity between temperatures T1 and T3 for FLN and FRN. Moreover, the plasticity QTLs *pfln1.1_T31_2E* and *pfln6.1_T31_2E* co-localized with *fln1.1_T1_2E* and *flnlog6.1_T3_2E* (Table 3; Supplementary Figure S1), likely reflecting the variation in those QTL effects due to the changes in temperature conditions.

The QTL analysis performed independently in each trial identified a total of 41 QTLs. Twenty-three QTLs were detected using non-transformed data. Fourteen were for FLN (9 in 2018 and 5 in 2019). The QTLs with more stable effects were localized in chromosome 2 at around 53–59 cM in 2018 and 2019 under T1 and T2, with the L5 allele increasing FLN. QTLs identified under T3 for FLN were exclusive of the temperature regime and were detected only in 2018, with *fln5.2_T3_18* in chromosome 5 explaining the highest level of phenotypic variance of 14.39% and the E9 allele increasing the trait. Seven QTLs were identified for FRN under T1 (in both years) and T2 (in 2019). For T2 in 2019, a QTL in chromosome 2, at the same position as for FLN, was identified, with the allele of L5 increasing the FRN and explaining 12.71% of the phenotypic variance for this trait. The number of QTLs identified for the FRS was low, with only two QTLs localized in chromosomes 9 and 7 in 2018 under T1 and T2, respectively. A QTL explaining 14.06% of the phenotypic variance was detected in chromosome 7, with the allele of L5 increasing the fruit set for the mild heat stress temperature T2 (Supplementary Table S2). Most of the QTLs detected independently for each year were also identified in the multi-environmental QTL analysis, except for the QTLs involved in FLN under T3, *fln9.1_T1_18*, *fln9.1_T1_18* and *fln1.1_T2_19,* while the QTLs associated with FRS were only detected in the independent analysis (Supplementary Table S2).

The QTLs detected in chromosomes 1, 2 and 5 were also identified with the log-transformed data. In addition, a new QTL affecting the FLN under T3 was detected in chromosome 6. Additionally, four new QTLs for FRN located in chromosomes 1, 2 and 5 were identified (Supplementary Table S2). Only three QTLs, *flnlog1.1_T2_19*, *frslog1.1_T3_19* and *frslog2.1_T3_19*, were detected exclusively in the single-year analysis. The rest of QTLs matched with those detected in the multi-environmental QTL analysis.

Five plasticity QTLs were detected for pFLN and pFRN (Supplementary Table S2). All of them were identified in the multi-environmental analysis as well.

Twenty-four QTLs (Supplementary Table S3) were detected by MQM analysis using MapQTL software for non-transformed data, analyzing each year separately. Eleven QTLs were detected in genomic regions previously associated with QTLs via ICIMaping analysis (Supplementary Table S3 and Supplementary Figure S2). However, 12 QTLs were detected exclusively with MapQTL, including three for FRS. Additionally, the allele effect QTL *frn11.1_T1_18* showed different directions between the two analyses.

#### 2.1.3. Epistatic Interactions in the RIL Population E9×L5

The epistatic analysis using the non-transformed data revealed fourteen pairs of significant epistatic QTLs (Table 4, Supplementary Table S4). For FLN and FRN, interactions were detected under T1, T2 and T3, while for FRS, interactions were detected only under T1. Six epistatic interactions were detected with the log-transformed data in the three temperatures regimes. Regarding the plasticity indexes, four interactions were detected, two for pFLN and two for pFRS (Table 4).

Four main effect QTLs mapped closely to epistatic genome regions, which could indicate that those QTLs were involved in the epistatic interactions, while for most of the epistatic regions, no QTLs were detected (Supplementary Figure S1). Interestingly, relatively high proportions of interaction for FRN and FRS were observed in the genomic region of 75–95 cM for chromosome 9, close to QTLs *frs9.1_T2_18, BCfln9.1_T1_2E* and *BCfln9.1_T2_2E* (Supplementary Table S4 and Supplementary Figure S3).

**Figure 2:**
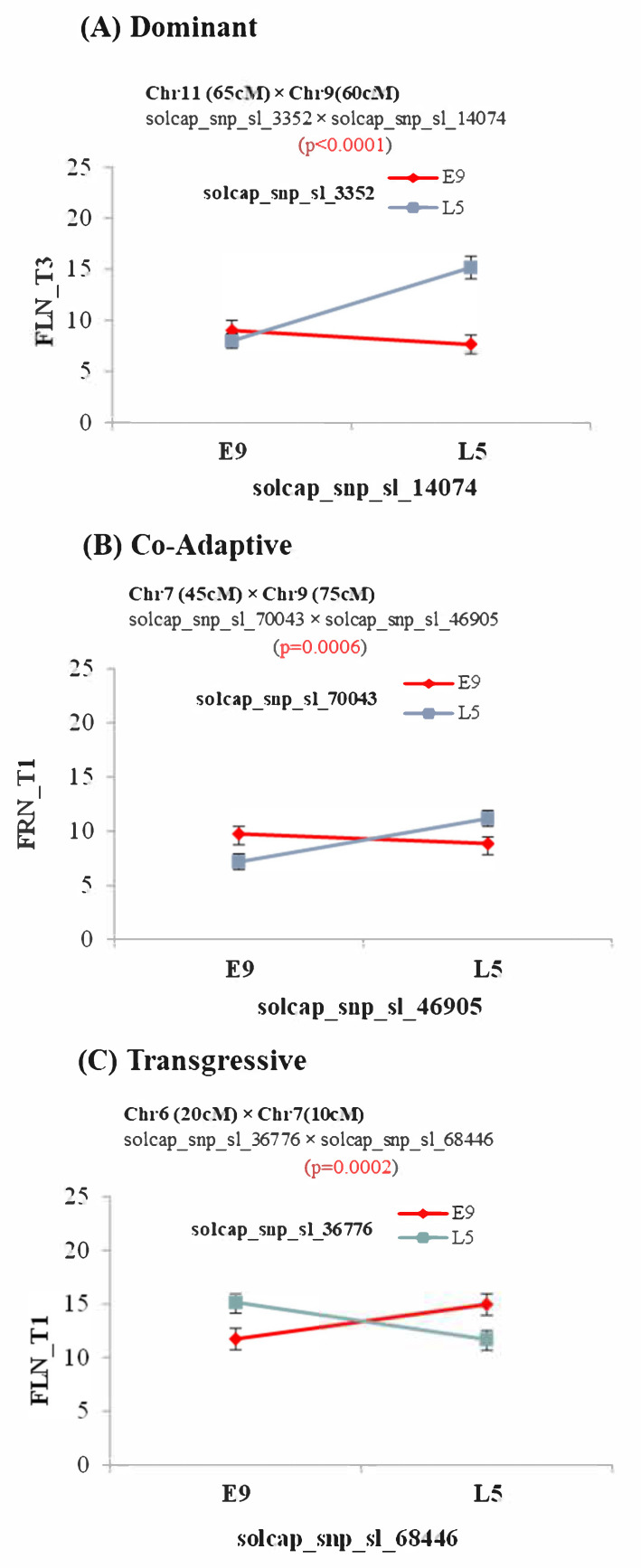
Examples of epistatic interaction patterns. The means of genotypic classes for the four possible homozygous allelic combinations (E9 and L5) are depicted for two pairs of markers showing significant epistatic interactions: (**A**) dominant epistasis for number of flowers under T3 (FLN_T3); (**B**) co-adaptive epistasis for number of fruits under T1 (FRN_T1); (**C**) transgressive epistasis for numbers of flowers under T1 (FLN_T1).

A two-way ANOVA was performed to verify the interactions and to obtain a biological interpretation of the epistasis [45]. All epistatic interactions detected were significant according to the ANOVA in at least one of the years in which the experiments were carried out, except for the interaction between regions, between chromosomes 1 and 2 for FLN_T1. Three types of epistasis were observed: dominant epistasis (one locus suppressed the allelic effects of a second locus; Figure 2A), co-adaptive epistasis (the double-homozygous alleles from the same parent showed a higher phenotypic value than the combination of homozygous alleles from alternative parents; Figure 2B) and transgressive epistasis (combination of double-homozygous alleles for alleles from different parents showed better performance than double-homozygous alleles from the same parent; Figure 2C). We have defined the last one as transgressive epistasis, because this type of interaction could be involved in the transgressive segregation in segregant populations. Dominant and transgressive epistases were the most common patterns (Supplementary Figure S3).

### 2.2. Inbred Backcross Line (IBL) Population E6203×LA1589

#### 2.2.1. Phenotypic Variations for Reproductive Traits at Different Temperatures

**Figure 3:**
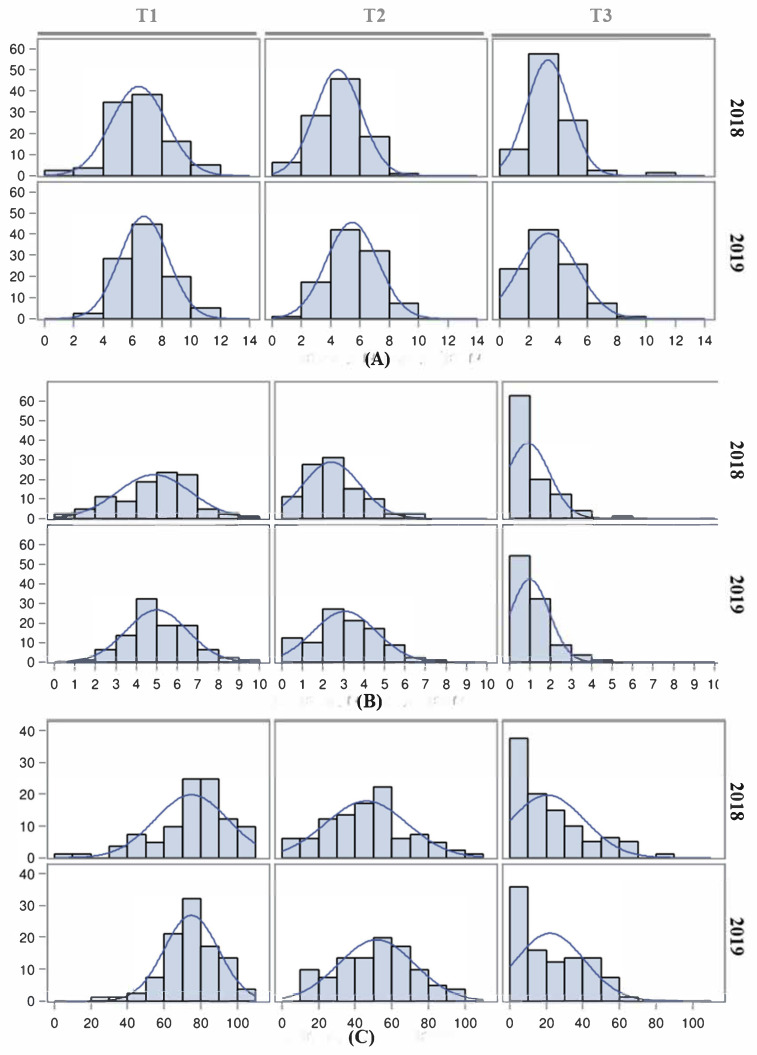
Histograms depicting the distribution of reproductive traits in the RIL population E6203×LA1589. (**A**) Flower number (FLN), (**B**) fruit number (FRN) and (**C**) fruit set (FRS) reproductive traits were studied in 2018 and 2019 for three temperature regimes (T1: 25 °C day/20 °C night; T2: 30 °C day/25 °C night; T3: 35 °C day/30 °C night).

Trait distributions for each temperature regime and year are represented in Figure 3. Means, standard deviations and ranges are described in Supplementary Table S1. The distribution of the reproductive traits was normal under T1 and T2, while a skewed distribution was observed under T3. FLN means and distributions under T1 and T2 were similar, with only a slight decrease in FLN under T2. At T3, the FLN decrease was most severe, with a reduction of 50% of the T3 FLN mean as compared with the T1 FLN mean, although most of the individuals in the population were able to generate flowers (Figure 3A).

For FRN, normal distributions were observed under T1 and T2. At T3, the fruit number was skewed to low values with a drastic FRN decrease (Figure 3B). Similarly, FRS was adversely affected by temperature, with an increasing reduction rate under T2 and T3 and a skewed distribution to low values under T3 (Figure 3C). The performances of the three traits were similar in both years.

Similarly to the observations in the E9×L5 population, the FRS trait showed a stronger decrease with the temperature increase. Nevertheless, high phenotypic variability was found in all traits and temperatures, suggesting that genetic variability may exist in this population for the heat stress response.

The correlation within and between years was analyzed for all reproductive traits. For FLN, the correlation was positive and highly significant at every temperature regime for both years (Table 5A). In 2018, no correlation was found for FRN under T1 or T2. However, a strong positive correlation was observed between T2 and T3 for this trait. In 2019, the correlation for FRN was significant and positive for the three temperatures regimes. The correlation between years showed a positive and significant correlation for all the temperatures as well (Table 5B).

For FRS, no correlation was observed either in 2018 or 2019 between temperatures, except under T1 and T2 in 2019, where a positive correlation was found. Moreover, FRS was not correlated between years for any of the temperature regimes (Table 5C).

The repeatability was higher under T1 for FLN, FRN and FRS for both non-transformed and log-transformed data (Table 6). Moreover, the values were similar for all three temperature regimes for FLN. However, the coefficient of repeatability decreased along with the temperature for the other traits. In general, the plasticity indexes showed low repeatability.

#### 2.2.2. Genetic Map

A total of 173,064 SNPs were obtained from the SPET genotyping procedure. After filtering, 4208 SNPs and 84 RILs were retained. Additionally, 177 markers with distorted segregation were excluded, with 4031 markers remaining for the linkage map generation (Supplementary Table S5). Linkage analysis identified 3297 SNPs (81.79%) with a recombination frequency equal to zero (0), while 68 SNPs (1.68%) not linked to any linkage group (LOD = 5.0) after the calculation of the first map. Thus, 3480 SNPs were excluded from the map (Supplementary Table S5), with 551 markers remaining for the generation of the final map.

The definitive map was constructed with only the 551 informative SNPs (45.9 SNPs/chromosome), resulting in a linkage map that spanned 1502 cM, with an average of 125 cM by linkage group, 1.6 SNPs/Mb, 0.4 SNPs/cM and 2.8 cM/SNP (Supplementary Table S5). The average distance between markers was 1581.0 kb, while the greatest distance between markers was 36,318.8 kb (Supplementary Figure S4). The marker order and distances on the map were as expected, with only 69 (1.71%) of the markers not collinear between their physical and genetic position. The specific information by chromosome is shown on Supplementary Tables S5 and S6.

#### 2.2.3. Identification of QTL in the IBL Population E6203×LA1589

In the multi-environment QTL analysis, a total of 16 QTLs were identified (Table 7). Seven QTLs were detected with the non-transformed data, four of which were for FLN under T1, T2 and T3. The *BCfln2.1_T3_2E* explained 15.27 % of the phenotypic variance, while the E6203 allele that increased the trait was identified under T3. QTLs for FRN and FRS were detected only under T1 (Table 7).

Six QTLs were detected with the log-transformed data, most of them also under T1. Interestingly, two QTLs were detected under T3 in chromosome 2, with the E6203 allele increasing the traits—*BCflnlog2.1_T3_2E* for LogFLN with R^2^ = 19.14%, coinciding with *BCfln2.1_T3_2E* and *BCfrnlog2.1_T3_2E* for LogFRN with R^2^ = 20.14%, which were not detected with the non-transformed data (Table 7).

Finally, three QTLs were identified for the plasticity indexes, two for pFLN and one for pFRS (Table 7). Here, *pBCfln2.1_T31_2E* co-localized with *BCfln2.1_T3_2E,* which may reflect the variation in genetics effects of this QTL induced by the temperature increase. The other two plasticity QTLs did not co-localize with any of the main effect QTLs.

The QTL analyses performed independently each year identified 13 QTLs for the non-transformed (7 QTLs), log-transformed data (3 QTLs, and plasticity indexes (3 QTLs) (Supplementary Table S7). Most of them coincided with the QTLs detected by multi-environmental analysis. Among them, *BCfln2.1_T3_19* for FLN under T3 explained 14.71% of the phenotypic variance, with the E6203 allele increasing the trait at the extremely high temperature.

### 2.3. Comparison between E9×L5 and E6203×LA1589 Populations

#### 2.3.1. Repeatability

In both populations, the coefficient of repeatability for the non-transformed data showed that the traits with the highest broad-sense heritability were FLN and FRN under T1 and T2. The FLN also presented high repeatability under T3 (Table S2 and Table 6), in agreement with the data obtained in the correlation analysis (Table 1 and Table 5). The repeatability of plasticity indexes was low in general.

#### 2.3.2. QTL Analysis

More QTLs were detected in the E9×L5 RIL population than in the E6203×LA1589 IBL population, which may have been due to differences in genetic diversity for the studied traits between populations, but also due to differences in population size and genetic structure. Most of the QTLs identified in this work were exclusive of the population. Two FLN QTLs located in chromosome 2 (*flnlog2.1_T2_2E* and *BCflnlog2.1_T1_2E*) were identified in similar positions in both populations at different temperature regimes. However, the L5 allele of *flnlog2.1_T2_2E* increased the trait, while the E6203 allele increased for *BCflnlog2.1_T1_2E*. Additionally, two QTLs were detected in a similar genomic region on chromosome 10 in both populations (*flnlog10.1_T2_2E* and *BCflnlog10.1_T1_2E*), with the wild parent allele (L5 or LA1589) increasing the trait in both cases.

A FRN QTL located in chromosome 2 in a position between 41,605,351 bp and 47,650,959 bp was detected in both populations using both non-transformed (*frn2.1_T2_2E* under T2; R2 = 6 and *BCfrn2.1_T1_2E*; R2 = 18.97) and log-transformed data (*frnlog2.1_T1_2E* and *frnlog2.1_T2_2E*, explaining 8.56% and 9.62% of the phenotypic variation, respectively, as well as *BCfrnlog2.1_T1_2E* with R^2^ = 30.43). The wild relative allele increased the trait in the E9×L5 population, while the cultivated allele did so in the E6203×LA1589 population.

Very few QTLs were detected for FRS under T3. Consequently, no common QTLs were found. Regarding plasticity indexes, only *pfln1.1_T31_2E* and *pBCfln1.1_T21_2E* mapped closely in their respective populations.

## 3. Discussion

The threat of climate change has resulted in the urgent need to understand the genetic control of abiotic stress responses in order to overcome the effects of environmental changes. Identifying sources of genetic diversity for heat tolerance may contribute towards the development of new varieties that are better adapted to the new environmental conditions by offering novel solutions to the problem. In the present study, we studied the genetic control of reproductive traits under different temperature regimes in two tomato inbred line populations derived from crosses between cultivated and wild relative accessions to search for genetic variability that may be useful for the development of new cultivars that might adapt to changing environmental conditions.

The effect of high temperatures on reproductive traits is evident when night temperatures are maintained over 25 °C [46], which may be frequently reached in the Mediterranean basin fields during summer. Thus, both inbred line populations were subjected to three temperature regimes (control T1, moderate stress T2 and extreme stress T3) to evaluate their responses to temperature changes and to identify QTLs involved in these responses. The effect of heat stress on FLN was more evident under T3, although the magnitude was not as drastic as reported previously [22,23,24,46,47], especially in the E9×L5 population, suggesting a better performance of this population under heat stress for this trait.

In general, both populations showed a good response to moderate heat stress under T2, with an average FRS close to 50% but with a wider range compared with T1. However, FRS was dramatically reduced under T3, with a low proportion of inbred lines being capable of setting fruits at this temperature, as reported previously [21,22,23,24,46,47,48]. The current results confirm that the heat stress mostly affects the fertility of the flowers and not their production. The lack of parents, due to different agronomical problems, made the confirmation of transgressive segregation impossible, although the wide range of values obtained under T2 and the extreme values under T3 suggest the existence of transgressive phenotypes, which are commonly reported in crosses of cultivated tomatoes with exotic germplasm [49,50,51]. The variability of responses under heat stress observed in both populations confirmed the complexity of the trait.

The strong and positive correlations, and the relatively high repeatability observed for FLN and FRN between the 2018 and 2019 under T1 and T2 experiments, indicated a significant hereditability of the tolerance under moderate heat stress. The correlation and repeatability of FLN and FRN under T3 were in general low and non-significant, suggesting no or low genetic variance for these traits at that heat stress. On the other hand, the low repeatability for FRS indicates a low genetic variability for this trait in the studied populations.

The genetic map generated for the E6203×LA1589 population had a total length of 1502 cM, similar to other published maps for this species, ranging from 1091 cM [52] to 2023 cM [53]. Moreover, the collinearity between the physical and genetic maps was very high, validating the robustness of the map to be used in QTL analysis.

Most of the detected QTLs were associated with FLN and FRN under T1 and T2 temperature regimes, coinciding with the heritability estimated in those trait and temperature regime combinations. Therefore, the results obtained in the current report may have impacted those conditions. On the other hand, additional QTLs were detected using log-transformed data or MQM analysis, which were remarkable for FRS under T3 in the E9×L5 population. This observation reinforces the need to optimize the data analysis for such complex traits to obtain the maximum information and to not miss potentially valuable QTLs such as *frslog5.2_T3_2E.* This low QTL detection, as well as the modest single QTL effects at high temperature regimes, was concomitant with the low genetic variability estimated via repeatability analysis, as found in previous studies [21,22,24,37,38,39], reflecting the genetic complexity of the response to heat stress. Even though an important proportion of the QTLs were population-specific, as previously found [39], some genomic regions contained QTLs clusters detected in several studies. Thus, FRN QTLs were detected in both E9×L5 and E6203×LA1589 at the bottom of chromosome 1. Additionally, IL SP_1–4, which harbors an introgression from the *S. pimpinellifolium* TO-937 accession on the same genomic region in the genetic background of the Moneymaker cultivar, very consistently showed high FRS and FRN under T3 in previous works [22], while a plasticity QTL for FRN also mapped to that region [39]. Another interesting QTL cluster was found at the bottom of chromosome 2, whereby *BCfln2.1_T3_2E* and FLN plasticity QTLs (in the population E6203×LA1589) co-located with FLN QTLs were detected in the Moneymaker×TO937 RIL population [22], GWAS studies [24,54] and MAGIC populations [39]. Another QTL cluster was found in the central region of chromosome 2, with QTLs involved in reproductive traits in several populations, for example, *fln2.1_T2_2E* (E9×L5), *BCfrn2.1_T1_2E* (E6203×LA1589), *fln2.1_T3_2E* (Moneymaker×TO-937, [22]), *nflw2.2*, *nflw2.3* (GWAS panel, [39]) and *nflw2* (MAGIC, [39]). These results suggest that those genomic regions are strong candidates for harboring useful genetic diversity for the study of the responses of reproductive traits under different temperature regimes. Other examples of co-localization include *flnlog6.1_T3_2E* and *flnlog10.1_T2_2E*, with QTLs identified in Moneymaker×TO-937 [22], *fln5.1_T2_2E* with nflw5.1 in the MAGIC population [39] and *fln2.1_T2_2E*, *pfln6.1_T31_2E* and *BCfln9.1_T2_2E* with associations from a GWAS panel [39]. Furthermore, the unexpected differences found in the QTL detection depending on the software could be due to differences in sensitivity to deviations from normality, as well as in the tools and algorithms used in each software for the selection of cofactors during multiple QTL mapping or composite interval mapping procedures, although simulation studies are needed for confirmation. In any case, the IciMapping software allows for multi-environmental QTL analysis, and in fact was able to detect 6 QTLs that were not detected at all when analyzing the years separately.

Bineau et al. [39] demonstrated the power of multiple parent populations in tomato plants to identify QTLs involved in response to heat stress. Compared with bi-parental population experiments (the current study and [22]), some similar conclusions can be drawn, i.e., a large proportion of the QTLs were population-specific, and the individual effects of the QTLs were usually modest. Nevertheless, some QTLs seem to be mapped in the same regions among populations, which could represent major adaptation QTLs and targets for further investigations. Thus, these results confirm the need to continuously search for new genetic variability for stress tolerance to obtain a global picture of the QTLs involved in heat stress to identify those with robust effects that could be integrated efficiently in breeding programs and to identify the underlying genes.

Alleles from both parents were found to be associated with tolerance to heat stress in the current and previous studies [22,37,38]. In the current report, wild alleles usually increased the traits in the E9×L5 population, while in the E6203×LA1589 population cultivated alleles were more frequently associated with a tolerant response in the reproductive trait. This variation in the direction of the additive effects, even within populations, might indicate that most of the studied accessions included alleles that may induce a positive response to heat stress, complementary to the alleles included in other accessions.

Interestingly, a relatively large number of epistatic interactions were detected in the E9×L5 population. Most of the interactions occurred in genomic regions that did not include any main effect QTL. The detection of epistatic interaction between QTLs is truly a challenge due to the low statistical power of the common experimental designs to detect these interactions [55]. The E9×L5 population had a balanced allelic frequency (f(L5) = f(E9) = 0.5), whereas in the E6203×LA1589 population, the allelic frequencies were unbalanced (f(E6203) = 0.875, f(LA1589) = 0.125). The power to detect epistasis is maximal in balanced populations, and the unbalanced allele was certainly a factor that made the detection of interactions difficult in the E6203×LA1589 population. Thus, the possibility that the lack of detection was due to low statistical power in this population cannot be ruled out. Nevertheless, the high number of epistatic interactions in the E9×L5 population contrasts with the low number of detected main effect QTLs. Remarkably, epistatic interactions were detected under T3 for FRN, but no main effect QTLs were detected at this temperature regime. This result suggests that an important proportion of the genetic variability for response to heat stress is non-additive in the E9×L5 population. This may also explain the relatively low number of detected QTLs under high temperature regimes, as the effects of the QTL alleles would depend on the allelic state of another locus at different chromosomes, so the main QTL effects may seem low or null when estimating single QTL effects, especially in cross-interactions, reinforcing the role of QTL interactions in important agronomic traits. Different types of epistasis were found, and transgressive epitasis, defined when the combination of double-homozygous alleles from different parents showed better performance than double-homozygous alleles from the same parent alleles (Figure 2C and Supplementary Figure S3), was interestingly frequent. This kind of interaction could be explained at the molecular level as interactions involving repressors or negative regulators that do not fit between species.

In summary, a low number of QTLs was found for each temperature regime and reproductive trait in both RIL populations. Among them, QTLs involved in FLN and FRN under optimal and moderate–high temperature regimes (T1 and T2) were mainly detected, meaning research must subscribe to these conditions. Low QTL detection rates in studies on heat tolerance of tomato were observed in previous works [22,24,37,38], corroborating the genetic complexity of heat tolerance in this crop. Studies involving multiple parent populations have been demonstrated to be very powerful for the detection of a larger number of QTLs, although the individual contribution of each QTL is relatively modest [39]. The search for new sources of tolerance is still needed to enhance our knowledge on the genetic control of this abiotic stress. In the current report, the role of non-additive genetic variability was reinforced, undoubtedly complicating the practical application of the scientific results. Despite the low number of QTLs, four genomic regions presented common QTLs among the two studied populations. One region on chromosome 1 and two regions on chromosome 2 were also previously associated with heat tolerance [22,24,39,54], while two others on chromosomes 6 and 10 were also validated in [22]. The concurrent identification of QTLs associated with reproductive traits under heat stress in the same localization area in different studies reinforces the importance of these regions in the adaptive response of reproductive traits to high temperatures in tomato plants.

## 4. Materials and Methods

### 4.1. Plant Material and Growing Conditions

Two populations of inbred lines were studied: E9×L5 consisting of 128 RILs (Recombinant Inbred Lines) derived by single-seed descent from the cross between *Solanum lycopersicum* var. cerasiforme line E9 × *S. pimpinellifolium* line L5, used extensively for salt tolerance analysis [56,57], and E6203×LA1589, consisting of 91 inbred backcross lines (IBLs) originated via single-seed descent to create a set of homozygous BC2F6 lines from the cross *S. lycopersicum* E6203 (processing tomato) × *S. pimpinellifolium* LA1589 [58]. The set of 91 BILs was a selection of the original set of 196 BILs used to maximize recombination frequency and mapping resolution. The 91 BILs were obtained from the Tomato Genetics Resource Center (Davis, USA). Three of the BILs did not germinate, so the final mapping population consisted of 88 BILs.

Both populations were cultivated in a glasshouse under semi-controlled temperature conditions in the Centro de Experiencias Cajamar (FCCV; Paiporta, Spain) facilities during 2018 and 2019. One replicate with three plants per RIL (in the same grow bag, only one RIL per bag) were randomly distributed in the greenhouse. Plants were grown with stepwise temperature increases according to [22] under three temperature regimes: T1: 25 °C day/20 °C night; T2: 30 °C day/25 °C night; T3: 35 °C day/30 °C night. Seedlings were transplanted to the greenhouse with 2–3 true leaves after about a month in the nursery. Plants were cultivated on hydroponic grow bags with controlled fertirrigation. After two weeks of acclimation, the temperature regime was set to T1. The numbers of flowers and fruits assessed over four weeks under T1 were recorded in the second and third truss areas (see below). Then, flowers, and fruits were pruned and the T2 regime was applied for 4 weeks. Again, plants were pruned prior to applying the T3 regime.

### 4.2. Phenotypic Evaluation

Phenotypic evaluations were performed in the same plants in the three temperature regimes after pruning prior to a regime change (see above). During the third week of each temperature regime, the number of flowers (FLN) was recorded in the corresponding second and third truss areas developed after the temperature regime change. The number of set fruit (FRN) was recorded on the fourth week. The fruit set (FRS) was calculated as FRS = 100 *FRN/FLN. All data were transformed using the logarithmic function to reduce the skewness. Phenotypic plasticity, i.e., the ability of a genotype to express a different phenotype depending on environmental variability, was also studied among different temperature regimes with the phenotypic plasticity indexes [39]: pT21 = ((T2 value − T1 value)/T1); pT31= ((T3 value − T1 value)/T1); pT32= ((T3value − T2 value)/T2).

### 4.3. Statistical Analysis

The basic statistics (mean, standard deviation, maximum and minimum values) and Pearson correlations were calculated with the JMP 12.1.0 software package (SAS Institute, Cary, NC, USA) [59]. Trait distributions were calculated with the R statistical software.

The repeatability of a trait was estimated as the correlation between two or more measures of that character in the same individual. In this case, the repeatability set an upper limit to the degree of genetic determination (broad sense heritability) and to the heritability (narrow sense heritability) [44]. Here, the repeatability coefficient, estimated for the values of each trait at the two consecutive years, was obtained from the ANOVA with two sources of variation (genotype and environment (years)) and calculated according to the following model:

*Y_ijk_ = µ + α_i_ + βj +εij*

where *Y_ij_* is the phenotypic trait value from *i*th genotype in *j*th environment; *µ* is the general average; *α_i_* is the effect of the first variation source (genotype); *β_j_* is the effect of the second variation source (environment = year); *εij* is the experimental error:

(1) $$\;\rho =\frac{\sigma_{P}^{2}}{\sigma_{P}^{2}+\sigma_{es}^{2}}=\frac{\sigma_{g}^{2}+\sigma_{eg}^{2}}{{(\sigma }_{g}^{2}+\sigma_{eg}^{2})+\sigma_{es}^{2}}=\frac{\sigma_{a}^{2}+\sigma_{d}^{2}+\sigma_{eg}^{2}}{{(\sigma }_{a}^{2}+\sigma_{d}^{2}+\sigma_{eg}^{2})+\sigma_{es}^{2}}\;\sim \;H^{2}$$

(2) $${\;\sigma }_{p}^{2}\;=\frac{\mathrm{MSgen}\;-\mathrm{MSerror}}{n.\;\mathrm{years}}\;{\;\sigma }_{es}^{2}\;=\;\mathrm{MSerror}$$

(3) $$\rho =\frac{\sigma_{P}^{2}}{\sigma_{P}^{2}+\sigma_{es}^{2}}$$

where $\sigma_{g}^{2}$ is the genetic variance between genotypes; $\sigma_{eg}^{2}$ is the general environmental variance (permanent effect) because there is an intrinsic environmental effect on each genotype, which occurs in the year or cultivated environment (source of variance between genotypes in population); and $\sigma_{es}^{2}$ is the special environmental variance (variance for time or location effect), defined as an effect intrinsic to the environmental fluctuation conditions between years or different environments (sources of variance in the same genotypes between years/environments). The phenotypic variance ($\sigma_{p}^{2}$), which is the sum of $\sigma_{g}^{2}+\sigma_{eg}^{2}$, was obtained from the mean squares from the ANOVA according to Equation (1), and was calculated using (MSgenotype − MSerror)/number of years, Equation (2)). A special environmental variance ($\sigma_{es}^{2}$) corresponds to MSerror. Then, the repeatability (*ρ*) was calculated as the ratio of the phenotypic variance to the sum of phenotypic variance and the special environmental variance (Equation (3)) [44,60,61].

### 4.4. Genetic Map

The E9×L5 RIL map was previously generated in [62] using the SolCAP tomato panel [63] and contained 1901 SNPs. The E6203×LA1589 map was generated de novo. DNA samples from young leaves of BCRIL were obtained according to [64], with slight modifications for extraction in 96-well plates [65]. IBLs were genotyped with the GP2SOL SNP array using Single Primer Enrichment Technology (SPET) [66] by IGA Technology Services (Udine, Italy). The initial SNP matrix was filtered using the TASSEL v. 5 software [67], discarding loci with minor allele frequency (MAF) ≤ 0.05 and missing data higher than 10%, genotypes with missing data ≥ 30% and loci with missing data or that were heterozygous in any of the parent genotypes. Moreover, segregation distortion was assessed via Chi-square test from the expected 0.875:0.125 ratio (for E6203 and LA1589 alleles, respectively) appropriate for BC2F7 generation at F7. The Bonferroni correction was used to adjust the *p*-values, resulting in an overall *α* level of 0.05/total SNPs [68].

The software QTL IciMapping Version 4 was used for map construction [69]. For linkage mapping, the software parameters “Mapping Population Type 11” and “Marker Space Type 1 for Intervals” (P1BC2RIL) were selected, with the genotypic frequency model A/B (0.8750:0.1250). The analyses were carried out separately by chromosome, as the physical position of each marker was previously known after alignment of SNPs using the SL2.5 version of the tomato genome as the reference. The linkage was analyzed with an LOD threshold of 3.0 and a recombination frequency threshold of 0.30. The RECORD (The Recombination Counting and Ordering) option was used for marker ordering, and to ripple the marker order, the SAD (sum of adjacent criterion) parameter was implemented [70]. In a second stage, after excluding SNPs according to the criteria mentioned above, a new map assembly was performed by changing the marker ordering parameter to “nnTwoOpt” and the LOD threshold to 5.0. Mapping distances were calculated with the Kosambi function [71]. The linkage map figure was plotted using the R [72] package LinkageMapView [73] and the QTL map using MapChart v. 2.3 [74].

### 4.5. QTL Analysis

The QTL analysis was conducted with non-transformed data, the logarithmically transformed data and relative variables. The multi-environment QTL analysis was performed with QTL IciMapping [69], with the ICIM-ADD procedure (Inclusive Composite Interval Mapping of Additive QTL). The LOD threshold was determined via a permutation test with 1000 resamplings with a type I error of 0.05. Likewise, the epistatic analysis was performed with ICIM-EPI by 1000 permutations and a type I error of 0.05. The interactions between chromosome regions were double-checked with a two-way ANOVA for marker pairs with significant interactions according to the IciMapping (ICIM) analysis. The analysis was performed with JMP 12.1.0 (SAS Institute, Cary, NC, USA).

Additionally, QTL analysis was performed independently each year by also using QTL IciMapping with the ICIM-ADD procedure (Inclusive Composite Interval Mapping of Additive (and Dominant) QTLs). QTLs for single experiments were named using an abbreviation of the trait, followed by the chromosome number, the QTL number within the chromosome and the temperature regime (T1, T2 or T3), adding a suffix with the experiment year. Moreover, the multiple-QTL mapping (MQM) procedure was carried out using the MapQTL^®^ 6 software [75]. A 5% genome-wide significance level was assessed via permutation tests. The corresponding critical LOD values ranged from 3.2 to 3.4 depending on the trait.

Multi-environment QTLs were named using an abbreviation of the trait, followed by the chromosome number, the QTL number within chromosome, the temperature regime (T1, T2 or T3) and the suffix _2E (indicating two years). QTLs for the E6203×LA1589 population were preceded by the BC abbreviation to differentiate them from the rest of the population.

The QTL positions were represented graphically using MapChart v. 2.3 [74].

## Figures and Tables

**Table 1:** Linear correlations in the E9×L5 population between the (**A**) FLN (flower number), (**B**) FRN (fruit number) and (**C**) FRS (fruit set) traits by year (2018 and 2019) and the three temperature regimes (T1: 25 °C day/20 °C night; T2: 30 °C day/25 °C night; T3: 35 °C day/30 °C night). Note: ** *p* < 0.01; * *p* < 0.05. The correlations within years are highlighted in grey, while the correlations between years are framed in orange.

**(A)**	**18FLN_T1**	**18FLN_T2**	**18FLN_T3**	**19FLN_T1**	**19FLN_T2**
**18FLN_T2**	0.37 **				
**18FLN_T3**	0.31 **	0.63 **			
**19FLN_T1**	0.56 **	0.30 **	0.23 *		
**19FLN_T2**	0.55 **	0.40 **	0.25 **	0.63 **	
**19FLN_T3**	0.40 **	0.39 **	0.24 **	0.47 **	0.47 **
**(B)**	**18FRN_T1**	**18FRN_T2**	**18FRN_T3**	**19FRN_T1**	**19FRN_T2**
**18FRN_T2**	0.44 **				
**18FRN_T3**	0.17	0.07			
**19FRN_T1**	0.54 **	0.36 **	0.05		
**19FRN_T2**	0.40 **	0.39 **	0.12	0.52 **	
**19FRN_T3**	0.24 *	0.13	0.19 *	0.33 **	0.39 **
**(C)**	**18FRS_T1**	**18FRS_T2**	**18FRS_T3**	**19FRS_T1**	**19FRS_T2**
**18FRS_T2**	0.02				
**18FRS_T3**	0.03	0.07			
**19FRS_T1**	0.15	0.19 *	0.03		
**19FRS_T2**	0.15	0.03	−0.11	0.39 **	
**19FRS_T3**	0.14	−0.15	0.21 *	0.20 *	0.34 *

**Table 2:** Coefficients of repeatability for the reproductive traits (FLN, FRN and FRS) and non-transformed data, log-transformed data and plasticity indexes for the E9×L5 population.

Repeatability Coefficient					
Non-Transformed		Log-Transformed		Plasticity Indexes	
**FLN_T1**	0.58	**LogFLN_T1**	0.53	**pFLN_T21**	0.10
**FLN_T2**	0.47	**LogFLN_T2**	0.43	**pFLN_T31**	0.18
**FLN_T3**	0.33	**LogFLN_T3**	0.24	**pFLN_T32**	0.12
**FRN_T1**	0.57	**LogFRN_T1**	0.56	**pFRN_T21**	0.08
**FRN_T2**	0.40	**LogFRN_T2**	0.42	**pFRN_T31**	0.09
**FRN_T3**	0.07	**LogFRN_T3**	0	**pFRN_T32**	0
**FRS_T1**	0.15	**LogFRS_T1**	0	**pFRS_T21**	0
**FRS_T2**	0.06	**LogFRS_T2**	0.14	**pFRS_T31**	0.18
**FRS_T3**	0.17	**LogFRS_T3**	0.17	**pFRS_T32**	0.06

**Table 3:** QTLs detected for the reproductive flower number (FLN), fruit number (FRN) and fruit set (FRS) traits, along with non-transformed data, log-transformed data and plasticity indexes at different temperature regimes (T1: 25 °C day/20 °C night; T2: 30 ° C day/25 °C night; T3: 35 °C day/30 °C night) in two experiments (2018 and 2019) as assessed by multi-environment QTL analysis with ICIMapping. QTLs are named using the trait abbreviation, followed by the chromosome number, the number of QTLs within the chromosome, the temperature regime and the suffix _2E. Plasticity QTLs are indicated by the *p* prefix. The estimates for each QTL include: maximum LOD score for genetic effects (LOD), LOD score of additive effects (LOD (A)), LOD score for the interactions of additive effects with the environment (LOD (AbyE)), percentages of phenotypic variance explained by the QTL (PVE) and additive effects (A), interactions of additives with the environment (AbyE) and the additive value (Add), which was negative when L5 alleles increased the trait and positive when E9 alleles increased it.

Trait	Temp.	QTL Name	Chr.	Position (cM)	Physical Position	Left Marker	Right Marker	LOD	LOD (A)	LOD (AbyE)	PVE	PVE (A)	PVE (AbyE)	Add
**FLN**	T1	*fln1.1_T1_2E*	1	48	68,120,455	solcap_snp_sl_50576	solcap_snp_sl_50571	8.87	8.53	0.34	21.64	16.4	5.23	−1.76
		*fln1.2_T1_2E*	1	87	77,186,438	solcap_snp_sl_34622	solcap_snp_sl_27832	4.86	2.56	2.3	6.9	4.71	2.19	−0.9
		*fln11.1_T1_2E*	11	82	50,661,076	solcap_snp_sl_100998	solcap_snp_sl_56295	4.24	1.57	2.67	5.85	2.87	2.98	−0.71
	T2	*fln1.1_T2_2E*	1	132	85,588,938	solcap_snp_sl_40586	solcap_snp_sl_40595	4.53	2.43	2.1	3.52	3.43	0.09	−1.28
		*fln2.1_T2_2E*	2	58	41,414,628	solcap_snp_sl_23884	solcap_snp_sl_29766	10.05	7.66	2.39	12.5	12.18	0.32	−2.48
		*fln5.1_T2_2E*	5	37	3,934,485	solcap_snp_sl_23738	solcap_snp_sl_23733	4.8	1.81	2.99	3.11	2.57	0.54	−1.1
**FRN**	T1	*frn1.1_T1_2E*	1	48	68,120,455	solcap_snp_sl_50576	solcap_snp_sl_50571	9.99	5.86	4.13	22.43	11.27	11.16	−0.98
		*frn1.2_T1_2E*	1	134	86,020,297	solcap_snp_sl_54525	solcap_snp_sl_54535	4.77	4.68	0.09	8.88	8.69	0.2	−0.82
		*frn4.1_T1_2E*	4	85	60,537,037	solcap_snp_sl_47056	CL017798-0849	4.23	2.93	1.3	8.62	5.5	3.12	−0.67
		*frn5.1_T1_2E*	5	47	4,941,314	solcap_snp_sl_48814	solcap_snp_sl_50687	4.72	3.16	1.56	9.15	5.98	3.17	−0.68
		*frn5.2_T1_2E*	5	75	61,030,288	solcap_snp_sl_22609	solcap_snp_sl_37191	4.66	2.71	1.96	9.54	5.06	4.47	−0.63
		*frn9.1_T1_2E*	9	39	4,450,858	solcap_snp_sl_39533	solcap_snp_sl_39517	4.22	2.11	2.11	8.14	4.03	4.12	−0.56
		*frn11.1_T1_2E*	11	82	50,661,076	solcap_snp_sl_100998	solcap_snp_sl_56295	4.23	1.85	2.38	8.13	3.49	4.63	−0.52
	T2	*frn1.1_T2_2E*	1	48	68,120,455	solcap_snp_sl_50576	solcap_snp_sl_50571	4.12	3.87	0.25	6.66	6.31	0.35	−1.47
		*frn2.1_T2_2E*	2	57	41,022,238	solcap_snp_sl_100921	solcap_snp_sl_100559	4.75	3.67	1.08	6.2	6.17	0.03	−1.43
**LogFLN**	T1	*flnlog1.1_T1_2E*	1	48	68,120,455	solcap_snp_sl_50576	solcap_snp_sl_50571	9.25	8.83	0.42	16.69	15.05	1.64	−0.06
	T2	*flnlog2.1_T2_2E*	2	58	41,414,628	solcap_snp_sl_23884	solcap_snp_sl_29766	10.82	9.36	1.45	17.67	17.34	0.33	−0.08
		*flnlog5.1_T2_2E*	5	37	3,934,485	solcap_snp_sl_23738	solcap_snp_sl_23733	5.06	2.65	2.41	6.57	4.46	2.12	−0.04
		*flnlog10.1_T2_2E*	10	71	62,415,424	solcap_snp_sl_33804	solcap_snp_sl_33797	3.89	3.85	0.04	6.34	6.1	0.24	0.04
	T3	*flnlog6.1_T3_2E*	6	86	43,388,539	solcap_snp_sl_57203	solcap_snp_sl_57155	4.24	3.73	0.5	7.15	7.13	0.02	−0.06
**LogFRN**	T1	*frnlog1.1_T1_2E*	1	135	86,635,078	solcap_snp_sl_54535	solcap_snp_sl_31775	4.2	4.04	0.15	6.72	6.26	0.46	−0.05
		*frnlog2.1_T1_2E*	2	53	40,380,985	solcap_snp_sl_49669	solcap_snp_sl_49679	7.17	5.18	2	8.83	8.56	0.27	−0.05
		*frnlog5.1_T1_2E*	5	75	60,779,710	solcap_snp_sl_22609	solcap_snp_sl_37191	5.69	1.89	3.8	5.64	3.07	2.58	−0.03
	T2	*frnlog2.1_T2_2E*	2	62	45,664,881	solcap_snp_sl_42692	solcap_snp_sl_42678	5.02	4.4	0.62	10.96	9.62	1.34	−0.08
**LogFRS**	T3	*frslog5.1_T3_2E*	5	16	2,246,381	solcap_snp_sl_49182	solcap_snp_sl_49127	5.98	1.51	4.47	8.47	3.48	4.99	0.05
		*frslog5.2_T3_2E*	5	55	5,969,101	solcap_snp_sl_50819	solcap_snp_sl_5050	3.9	2.41	1.49	6	5.16	0.84	−0.05
**pFLN_T31**	T3 vs. T1	*pfln1.1_T31_2E*	1	56	71,079,273	solcap_snp_sl_38059	solcap_snp_sl_38078	5.01	3.02	1.98	8.7	6.27	2.43	0.1
	T3 vs. T1	*pfln6.1_T31_2E*	6	81	42,683,699	solcap_snp_sl_57294	solcap_snp_sl_57252	4.65	3.81	0.84	8.26	7.64	0.62	−0.11
**pFRN_T31**	T3 vs. T1	*pfrn2.1_T31_2E*	2	34	37,011,375	solcap_snp_sl_29543	solcap_snp_sl_13632	63.52	62.36	1.16	20.28	10.45	9.83	−0.33
	T3 vs. T1	*pfrn3.1_T31_2E*	3	122	63,813,612	solcap_snp_sl_33829	solcap_snp_sl_61238	4.29	3.78	0.51	0.37	0.27	0.1	0.05
**pFRN_T32**	T3 vs. T2	*pfrn5.1_T32_2E*	5	7	1,527,883	solcap_snp_sl_52738	solcap_snp_sl_52748	3.43	0.49	2.94	14.59	3.47	11.11	0.06

**Table 4:** Epistatic interactions in the E9×L5 population for non-transformed data, log-transformed data and plasticity traits. Reproductive trait, flower number (FLN), fruit number (FRN) and fruit set (FRS) values were studied in three different temperature regimes (T1: 25 °C day/20 °C night; T2: 30 °C day/25 °C night; T3: 35 °C day/30 °C night). A multi-environment QTL analysis was performed with ICIMapping. QTL1 and QTL2 columns indicate major effect QTLs identified in the E9×L5 and E6203×LA1589 population. The statistical significance (*p*-value) of the two-way ANOVA and the epistasis types (transgressive, co-adaptive and dominant) is also presented.

		Chr1/Position1		QTL1	Chr2/Position2												QTL2	ANOVA_18 *p*-Value	ANOVA-19 *p*-Value	
Trait	Temp	Chr	Pos (cM)		1	2	3	4	5	6	7	8	9	10	11	12				Epistasis Type
**FLN**	**T1**	1	85			20											*fln1.2_T2_2E*	ns	ns	-
		6	20								10							<0.0001	0.0008	transgressive
	**T2**	8	0											60				0.0001	ns	co-adaptive
		8	45										40				*fln9.1_T1_18*	0.0014	ns	dominant
		11	25													65		0.0005	0.0288	dominant
	**T3**	9	60	*BCfln9.1_T1*											65		*fln/frn11.1_T1*	<0.0001	ns	dominant
**FRN**	**T1**	7	45										75					0.0006	0.0005	co-adaptive
	**T2**	4	65											70			*frn10.1_T2_18*	0.0001	ns	dominant
		6	20								30							<0.0001	ns	transgressive
		7	60										95				*BCfln9.1_T2*	0.0008	ns	co-adaptive
		11	5													65	*frn11.1_T1_2E*	0.0025	ns	transgressive
	**T3**	3	105	*fln3.3_T1_19*											5			ns	<0.0001	transgressive
		7	95	*frs7.2_T2_18*										0				ns	<0.0001	transgressive
**FRS**	**T1**	3	120	*fln3.1_T1_19*									95				*BCfln9.1_T2*	0.0002	0.0003	transgressive
**Log FLN**	**T3**	8	100													105		<0.0001	ns	transgressive
**Log FRN**	**T1**	7	45										75					0.0009	0.0005	co-adaptive
	**T2**	7	60										95					<0.0001	ns	dominant
**Log FRS**	**T1**	3	115										90					0.0002	0.0015	dominant
	**T2**	2	40								5							ns	0.0001	dominant
	**T3**	5	20										25				*frslog5.1_T3_2E*	0.0002	ns	transgressive
**pFLN**	**T2 vs. T1**	8	0											60				<0.0001	ns	co-adaptive
	**T3 vs. T1**	7	35												0			<0.0001	ns	dominant
**pFRS**	**T3 vs. T2**	2	0			25												<0.0001	ns	dominant
		2	35					25										<0.0001	ns	dominant

**Table 5:** Linear correlations in the E6203×LA1589 population among (**A**) FLN (flower number), (**B**) FRN (fruit number) and (**C**) FRS (fruit set) for different experimental years (2018 and 2019) and temperature regimes (T1: 25 °C day/20 °C night; T2: 30 °C day/25 °C night; T3: 35 °C day/30 °C night). Note: ** *p* < 0.01; * *p* < 0.05. Significant correlations within years are highlighted in grey and correlations between years are framed in orange.

**(A)**	**18FLN_T1**	**18FLN_T2**	**18FLN_T3**	**19FLN_T1**	**19FLN_T2**
**18FLN_T2**	0.32 **				
**18FLN_T3**	0.42 **	0.57 **			
**19FLN_T1**	0.39 **	0.22	0.32 **		
**19FLN_T2**	0.23 *	0.34 **	0.28 *	0.51 **	
**19FLN_T3**	0.22	0.24 *	0.31 **	0.36 **	0.32 **
**(B)**	**18FRN_T1**	**18FRN_T2**	**18FRN_T3**	**19FRN_T1**	**19FRN_T2**
**18FRN_T2**	0.16				
**18FRN_T3**	0.07	0.33 **			
**19FRN_T1**	0.53 **	0.15	0.12		
**19FRN_T2**	0.34 **	0.31 **	0.23 *	0.52 **	
**19FRN_T3**	0.18	0.13	−0.04	0.25 *	0.23 **
**(C)**	**18FRS_T1**	**18FRS_T2**	**18FRS_T3**	**19FRS_T1**	**19FRS_T2**
**18FRS_T2**	0.06				
**18FRS_T3**	0.02	0.18			
**19FRS_T1**	0.18	0.06	−0.06		
**19FRS_T2**	0.17	0.09	0.27 *	0.29 **	
**19FRS_T3**	0.09	0.12	−0.01	0.01	0.07

**Table 6:** Coefficients of repeatability for the reproductive traits (FLN, FRN and FRS) for non-transformed data, log-transformed data and plasticity indexes for the E6203×LA1589 population.

Repeatability Coefficient					
Non-Transformed		Log-Transformed		Plasticity Traits	
**FLN_T1**	0.39	**LogFLN_T1**	0.37	**pFLN_T21**	0.17
**FLN_T2**	0.33	**LogFLN_T2**	0.31	**pFLN_T31**	0.23
**FLN_T3**	0.31	**LogFLN_T3**	0.36	**pFLN_T32**	0.15
**FRN_T1**	0.52	**LogFRN_T1**	0.51	**pFRN_T21**	0.07
**FRN_T2**	0.30	**LogFRN_T2**	0.30	**PFRN_T31**	0.00
**FRN_T3**	0.00	**LogFRN_T3**	0.08	**pFRN_T32**	0.00
**FRS_T1**	0.17	**LogFRS_T1**	0.10	**pFRS_T21**	0.00
**FRS_T2**	0.09	**LogFRS_T2**	0.03	**pFRS_T31**	0.00
**FRS_T3**	0.00	**LogFRS_T3**	0.00	**pFRS_T32**	0.00

**Table 7:** QTLs detected in the E6203×LA1589 population for the flower number (FLN), fruit number (FRN) and fruit set (FRS) reproductive traits with non-transformed data, log-transformed data and plasticity indexes at different temperature regimes (T1: 25 °C day/20 °C night; T2: 30 °C day/25 °C night; T3: 35 °C day/30 °C night) in two experiments (2018 and 2019) using multi-environment QTL analysis with ICIMapping. QTLs are named with the trait abbreviation, followed by the chromosome number, the number of QTLs within the chromosome, the temperature regime and the suffix _2E. The estimates for each QTL include: maximum LOD score for genetic effects (LOD), LOD score of additive effects (LOD (A)), LOD score for the interaction of additive effects with environment (LOD (AbyE)), percentages of phenotypic variance explained by the QTL (PVE) and additive effects (A), interaction additive by environment (AbyE) and the additive value (Add), being negative when LA1589 alleles increased the trait and positive when E6203 increased it.

Trait	Temp.	QTL Name	Chr.	Position (cM)	Physical Position	Left Marker	Right Marker	LOD	LOD (A)	LOD (AbyE)	PVE	PVE (A)	PVE (AbyE)	Add
**FLN**	T1	*BCfln9.1_T1_2E*	9	58	63,339,814	3945-CH09_63339814	3948-CH09_63457006	4.35	3.53	0.82	10.12	8.59	1.53	−0.75
		*BCfln10.1_T1_2E*	10	62	61,961,674	4437-CH10_61961674	4440-CH10_62144335	6.91	6.61	0.30	16.72	16.20	0.52	0.98
	T2	*BCfln9.1_T2_2E*	9	76	66,172,567	3998-CH09_66172567	4001-CH09_66360717	4.02	3.81	0.21	12.77	11.71	1.06	−0.89
	T3	*BCfln2.1_T3_2E*	2	111	50,499,308	1176-CH02_50499308	1184-CH02_51130940	4.96	4.78	0.18	15.27	14.15	1.12	0.87
**FRN**	T1	*BCfrn2.1_T1_2E*	2	68	41,605,351	1003-CH02_41605351	1026-CH02_43291158	9.32	7.84	1.48	22.48	18.97	3.51	1.31
		*BCfrn10.1_T1_2E*	10	37	4,411,465	4225-CH10_4411465	4304-CH10_44020893	4.68	3.62	1.06	10.06	8.44	1.62	0.54
**FRS**	T1	*BCfrs7.1_T1_2E*	7	0	1,345,511	2947-CH07_1345511	2975-CH07_2649973	8.47	8.32	0.15	27.82	23.90	3.92	21.01
**LogFLN**	T1	*BCflnlog2.1_T1_2E*	2	68	41,605,351	1003-CH02_41605351	1026-CH02_43291158	5.70	5.53	0.16	18.14	15.27	2.87	0.08
		*BCflnlog10.1_T1_2E*	10	62	61,961,674	4437-CH10_61961674	4440-CH10_62144335	7.00	6.87	0.13	20.03	20.00	0.03	0.07
	T3	*BCflnlog2.1_T3_2E*	2	111	50,499,308	1176-CH02_50499308	1184-CH02_51130940	6.74	6.68	0.06	19.41	17.21	2.19	0.17
**LogFRN**	T1	*BCfrnlog2.1_T1_2E*	2	68	41,605,351	1003-CH02_41605351	1026-CH02_43291158	11.80	11.64	0.16	38.08	30.43	7.65	0.18
	T3	*BCfrnlog2.1_T3_2E*	2	0	33,365,408	852-CH02_33365408	874-CH02_34472675	5.46	2.80	2.66	20.14	11.83	8.30	0.16
**LogFRS**	T1	*BCfrslog7.1_T1_2E*	7	0	1,345,511	2947-CH07_1345511	2975-CH07_2649973	12.64	12.61	0.03	74.01	49.13	24.87	0.22
**pFLN_T21**	T2 vs. T1	*pBCfln1.1_T21_2E*	1	42	76,264,322	282-CH01_76264322	292-CH01_77115304	31.04	29.91	1.13	39.12	21.70	17.42	0.67
**pFLN_T31**	T3 vs. T1	*pBCfln2.1_T31_2E*	2	111	50,499,308	1176-CH02_50499308	1184-CH02_51130940	4.90	4.89	0.01	19.12	17.70	1.43	0.14
**pFRS_T32**	T3 vs. T2	*pBCfrs2.1_T32_2E*	2	135	53,826,295	1255-CH02_53826295	1260-CH02_54014872	21.84	17.98	3.86	21.11	12.27	8.84	−1.24

## Data Availability

Data used for QTL analysis in the current report is available at https://doi.org/10.5281/zenodo.6457630, accessed on 22 March 2022.

## Supplementary Materials

The following are available online at https://www.mdpi.com/article/10.3390/plants11081069/s1, Figure S1: Map position of QTLs for non-transformed data, log-transformed data and plasticity indexes identified in E9×L5 and E6203×LA1589 populations by ICIMapping and MapQTL® 6 software. Additionally, QTLs identified in another RIL population from the cross between MoneyMaker × TO-937 [22] and the position of the epistatic interactions are indicated. Figure S2: Comparison of QTLs detected in E5xL9 and E6203×LA1589 populations using IciMapping for the two years combined (E9×L5 and E6203×LA1589), and using IciMapping and MapQTL software in the RIL population for each year separately (IcIE9×L5 and MapQTLE9×L5). The color of each QTL name indicates the type of QTL analysis. The chromosome, the physical position of QTL peaks in Mbp, additive values (add) and LOD scores are specified. Figure S3: Epistatic interactions for the (A) non-transformed data (B), log-transformed data and (C) plasticity indexes for flower number (FLN), fruit number (FRN) and fruit set (FRS) parameters under different temperature regimes (T1: 25 °C day/20 °C night; T2: 30 °C day/25 °C night; T3: 35 °C day/30 °C night). Supplementary Figure S4: Genetic linkage map of tomato plants, constructed based on the E6203×LA1589 IBL population with 551 SNPs. Table S1: Mean values, standard deviation and the maximum and minimum values for reproductive traits under three temperature regimes (T1: 25 °C day/20 °C night; T2: 30 °C day/25 °C night; T3: 35 °C day/30 °C night) in 2018 and 2019 for the E9×L5 and E6203×LA1589 populations. Table S2: QTLs detected in the E9×L5 population for the flower number (FLN), fruit number (FRN), and fruit set (FRS) reproductive traits, using non-transformed data, log-transformed data and the plasticity indexes under different temperature regimes (T1: 25 °C day/20 °C night; T2: 30 °C day/25 °C night; T3: 35 °C day/30 °C night) in two experiments (2018 and 2019) in an independent QTL analysis assessed by year with ICIMapping. QTLs are named using the trait abbreviation, followed by the chromosome number, the number of QTLs within the chromosome, the temperature regime and the experiment year. Plasticity QTLs are indicated by the p prefix. The estimates for each QTL include: maximum LOD score for genetic effects (LOD), phenotypic variance explained by the QTL (PVE) and the additive value (Add), being negative when L5 alleles increased the trait and positive when E9 alleles increased it. Common QTLs between independent analysis by year and multi-environment QTL analysis are highlighted in blue. Table S3: QTLs detected in the E9×L5 population for the reproductive traits flower number (FLN), fruit number (FRN) and fruit set (FRS) under different temperature regimes (T1: 25 °C day/20 °C night; T2: 30 °C day/25 °C night; T3: 35 °C day/30 °C night) in two experiments (2018 and 2019) via MQM analysis. QTLs detected exclusively with this analysis are highlighted in yellow. The statistical estimates for each QTL include: the chromosome position in the genetic map (cM) Maximum LOD score (LOD), percentage of phenotypic variance explained by the QTL (PVE) and the additive value (Add), being negative when L5 alleles increased the trait and positive when E9 increased it. Table S4: (A) Epistatic interaction in the E9×L5 population for the non-transformed data and log-transformed data for FLN, FRN and FRS and plasticity indexes. (B) Physical distances of the epistatic interactions. Table S5: Statistics for the genetic map from the E6203×LA1589 IBL population. Table S6: Name, position and characteristics of the SNPs used for the map generation. Table S7: QTLs detected in the E6203×LA1589 population for the flower number (FLN), fruit number (FRN) and fruit set (FRS) reproductive traits from non-transformed data, log-transformed data and the plasticity indexes under different temperature regimes (T1: 25 °C day/20 °C night; T2: 30 °C day/25 °C night; T3: 35 °C day/30 °C night) in two experiments (2018 and 2019) in an independent QTL analysis by year with ICIMapping. QTLs are named using the trait abbreviation, followed by the chromosome number, the number of QTLs within the chromosome, the temperature regime and the experiment year. Plasticity QTLs are indicated by the p prefix. The estimates for each QTL include: maximum LOD score for genetic effects (LOD), phenotypic variance explained by the QTL (PVE) and the additive value (Add), being negative when L5 alleles increased the trait and positive when E9 alleles increased it. Common QTLs between independent analysis by year and multi-environment QTL analysis are highlighted in blue.
