# Supplementary material for: Genetic Control of Reproductive Traits under Different Temperature Regimes in Inbred Line Populations Derived from Crosses between S. pimpinellifolium and S. lycopersicum Accessions
Source: Plants (Basel). 2022 Apr 14;11(8):1069. doi: 10.3390/plants11081069 (PMC9027731; doi:10.3390/plants11081069)
Supplement: Supplementary file 1 [file plants-11-01069-s001.zip › Supplem Figure S3.pptx]

## Slide 1
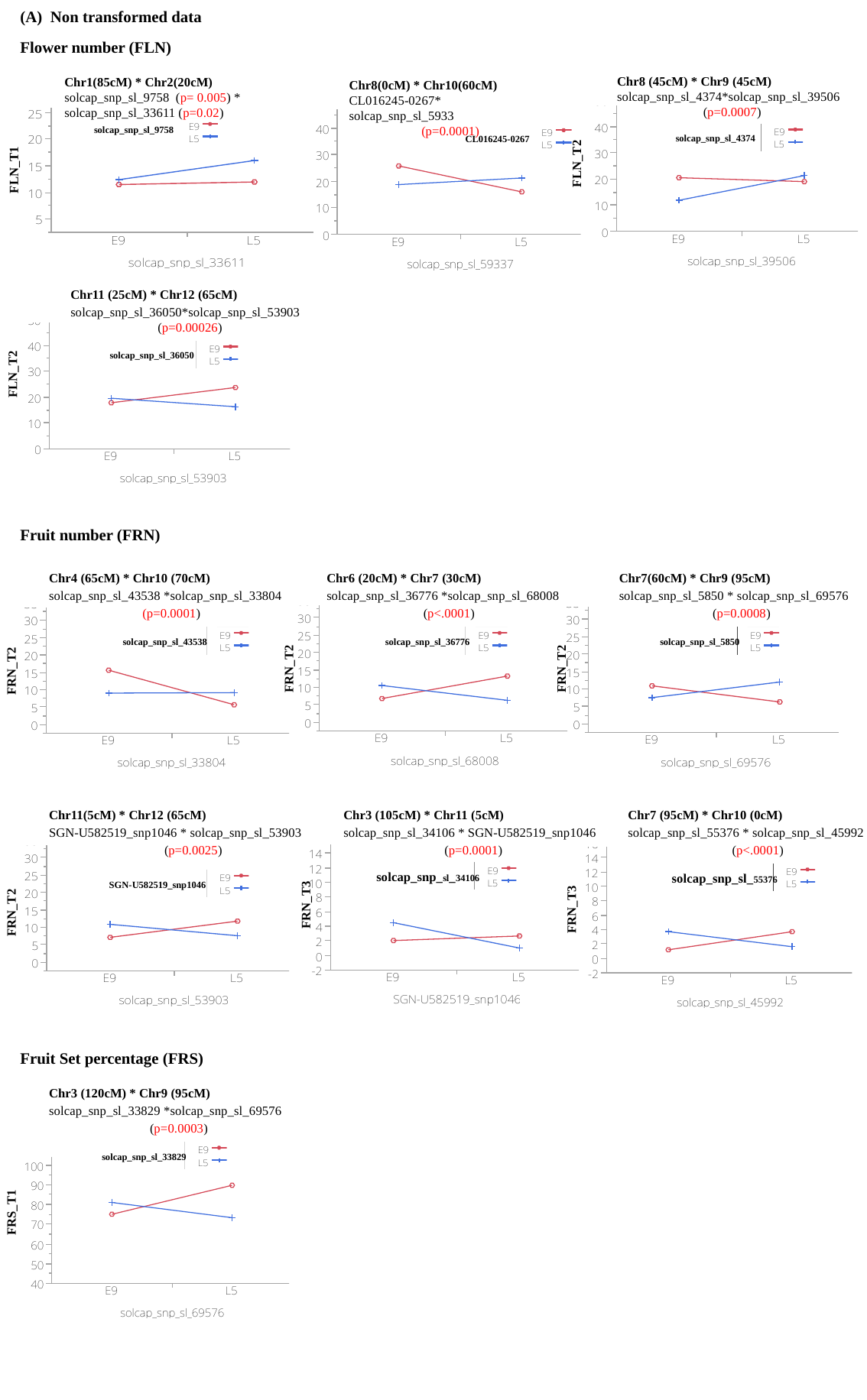

(A) Non transformed data
Flower number (FLN)
Chr8 (45cM) * Chr9 (45cM)
solcap_snp_sl_4374*solcap_snp_sl_39506
(p=0.0007)
Chr1(85cM) * Chr2(20cM)
solcap_snp_sl_9758 (p= 0.005) *
solcap_snp_sl_33611 (p=0.02)
Chr8(0cM) * Chr10(60cM)
CL016245-0267* solcap_snp_sl_5933
 (p=0.0001)
solcap_snp_sl_9758
solcap_snp_sl_4374
CL016245-0267
FLN_T2
FLN_T2
FLN_T1
Chr11 (25cM) * Chr12 (65cM)
solcap_snp_sl_36050*solcap_snp_sl_53903
(p=0.00026)
solcap_snp_sl_36050
FLN_T2
Fruit number (FRN)
Chr4 (65cM) * Chr10 (70cM)
solcap_snp_sl_43538 *solcap_snp_sl_33804
(p=0.0001)
Chr6 (20cM) * Chr7 (30cM)
solcap_snp_sl_36776 *solcap_snp_sl_68008
(p<.0001)
Chr7(60cM) * Chr9 (95cM)
solcap_snp_sl_5850 * solcap_snp_sl_69576
(p=0.0008)
solcap_snp_sl_43538
solcap_snp_sl_36776
solcap_snp_sl_5850
FRN_T2
FRN_T2
FRN_T2
Chr11(5cM) * Chr12 (65cM)
SGN-U582519_snp1046 * solcap_snp_sl_53903
(p=0.0025)
Chr3 (105cM) * Chr11 (5cM)
solcap_snp_sl_34106 * SGN-U582519_snp1046
(p=0.0001)
Chr7 (95cM) * Chr10 (0cM)
solcap_snp_sl_55376 * solcap_snp_sl_45992
(p<.0001)
solcap_snp_sl_34106
solcap_snp_sl_55376
SGN-U582519_snp1046
FRN_T3
FRN_T3
FRN_T2
Fruit Set percentage (FRS)
Chr3 (120cM) * Chr9 (95cM)
solcap_snp_sl_33829 *solcap_snp_sl_69576
(p=0.0003)
solcap_snp_sl_33829
FRS_T1

## Slide 2
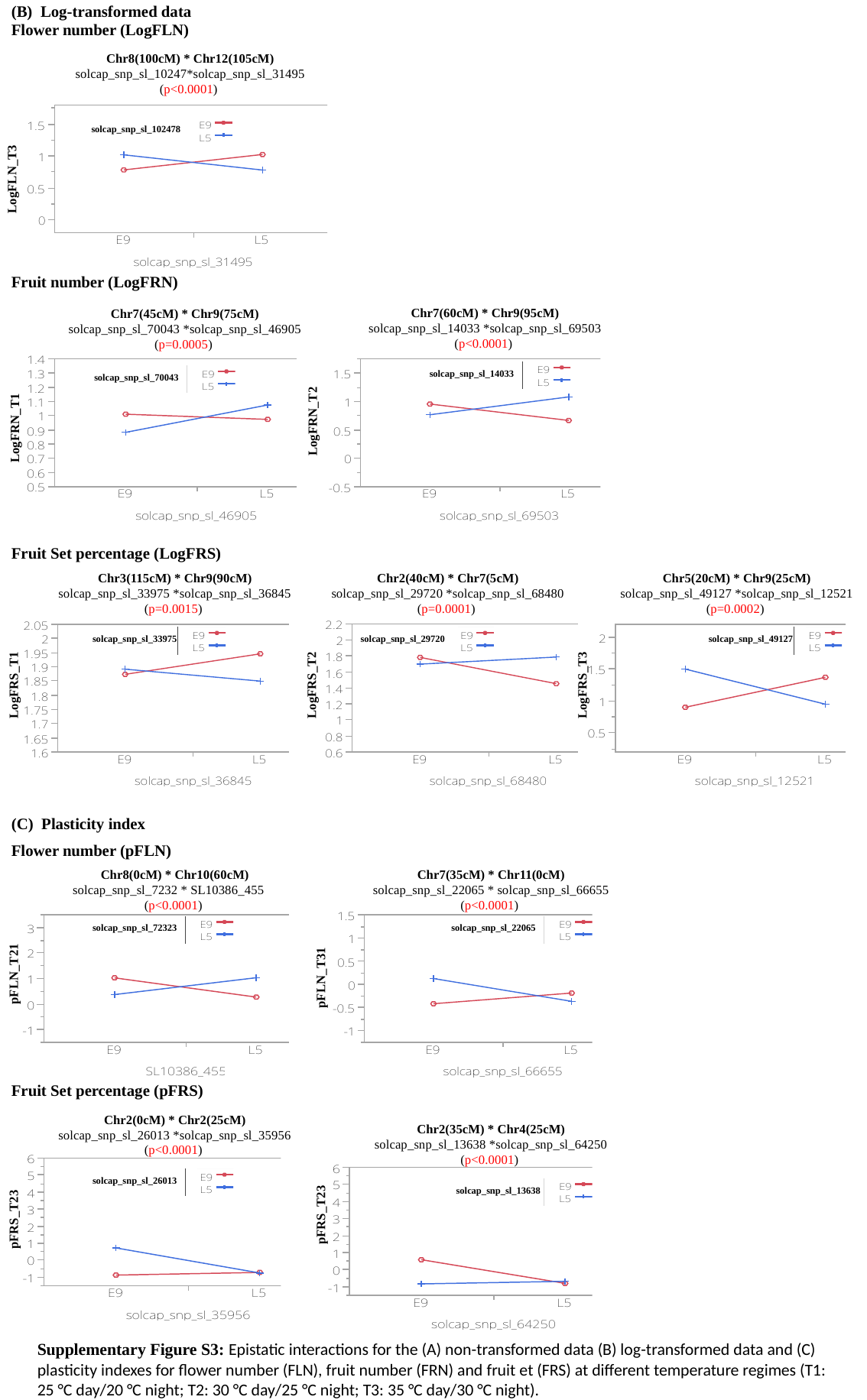

(B) Log-transformed data
Flower number (LogFLN)
Chr8(100cM) * Chr12(105cM)
solcap_snp_sl_10247*solcap_snp_sl_31495 (p<0.0001)
solcap_snp_sl_102478
LogFLN_T3
Fruit number (LogFRN)
Chr7(60cM) * Chr9(95cM)
solcap_snp_sl_14033 *solcap_snp_sl_69503 (p<0.0001)
Chr7(45cM) * Chr9(75cM)
solcap_snp_sl_70043 *solcap_snp_sl_46905 (p=0.0005)
solcap_snp_sl_14033
solcap_snp_sl_70043
LogFRN_T2
LogFRN_T1
Fruit Set percentage (LogFRS)
Chr3(115cM) * Chr9(90cM)
solcap_snp_sl_33975 *solcap_snp_sl_36845 (p=0.0015)
Chr2(40cM) * Chr7(5cM)
solcap_snp_sl_29720 *solcap_snp_sl_68480 (p=0.0001)
Chr5(20cM) * Chr9(25cM)
solcap_snp_sl_49127 *solcap_snp_sl_12521 (p=0.0002)
solcap_snp_sl_33975
solcap_snp_sl_29720
solcap_snp_sl_49127
LogFRS_T1
LogFRS_T2
LogFRS_T3
(C) Plasticity index
Flower number (pFLN)
Chr8(0cM) * Chr10(60cM)
solcap_snp_sl_7232 * SL10386_455 (p<0.0001)
Chr7(35cM) * Chr11(0cM)
solcap_snp_sl_22065 * solcap_snp_sl_66655 (p<0.0001)
solcap_snp_sl_72323
solcap_snp_sl_22065
pFLN_T21
pFLN_T31
Fruit Set percentage (pFRS)
Chr2(0cM) * Chr2(25cM)
solcap_snp_sl_26013 *solcap_snp_sl_35956 (p<0.0001)
Chr2(35cM) * Chr4(25cM)
solcap_snp_sl_13638 *solcap_snp_sl_64250 (p<0.0001)
solcap_snp_sl_26013
solcap_snp_sl_13638
pFRS_T23
pFRS_T23
Supplementary Figure S3: Epistatic interactions for the (A) non-transformed data (B) log-transformed data and (C) plasticity indexes for flower number (FLN), fruit number (FRN) and fruit et (FRS) at different temperature regimes (T1: 25 °C day/20 °C night; T2: 30 °C day/25 °C night; T3: 35 °C day/30 °C night).
