# Supplementary material for: Genetic Control of Reproductive Traits under Different Temperature Regimes in Inbred Line Populations Derived from Crosses between S. pimpinellifolium and S. lycopersicum Accessions
Source: Plants (Basel). 2022 Apr 14;11(8):1069. doi: 10.3390/plants11081069 (PMC9027731; doi:10.3390/plants11081069)
Supplement: Supplementary file 1 [file plants-11-01069-s001.zip › Supplem Figure S4.pptx]

## Slide 1
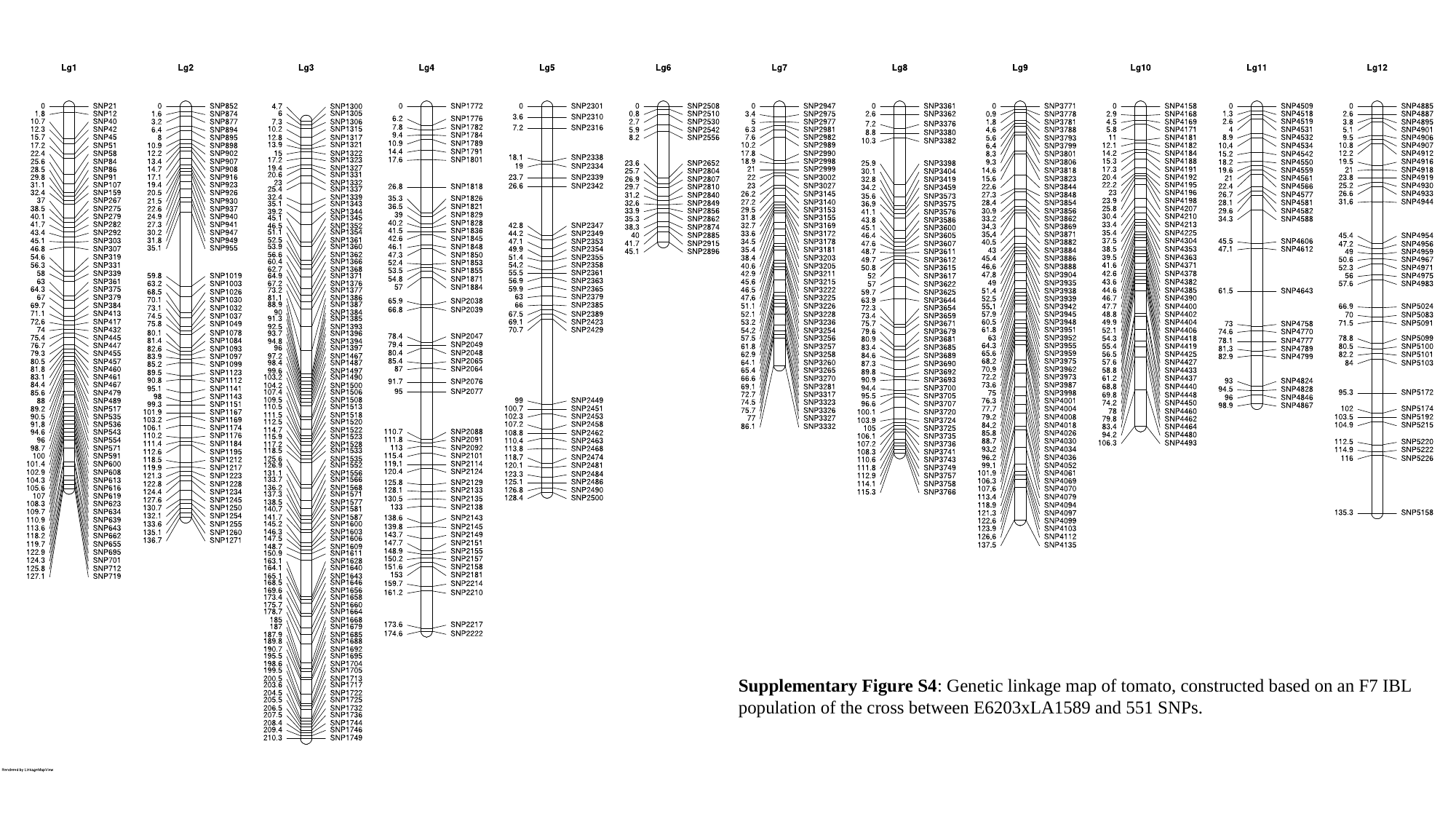

Supplementary Figure S4: Genetic linkage map of tomato, constructed based on an F7 IBL population of the cross between E6203xLA1589 and 551 SNPs.
