# Supplementary material for: Genetic Control of Reproductive Traits under Different Temperature Regimes in Inbred Line Populations Derived from Crosses between S. pimpinellifolium and S. lycopersicum Accessions
Source: Plants (Basel). 2022 Apr 14;11(8):1069. doi: 10.3390/plants11081069 (PMC9027731; doi:10.3390/plants11081069)
Supplement: Supplementary file 1 [file plants-11-01069-s001.zip › Supplementary Figure S1.pptx]

## Slide 1
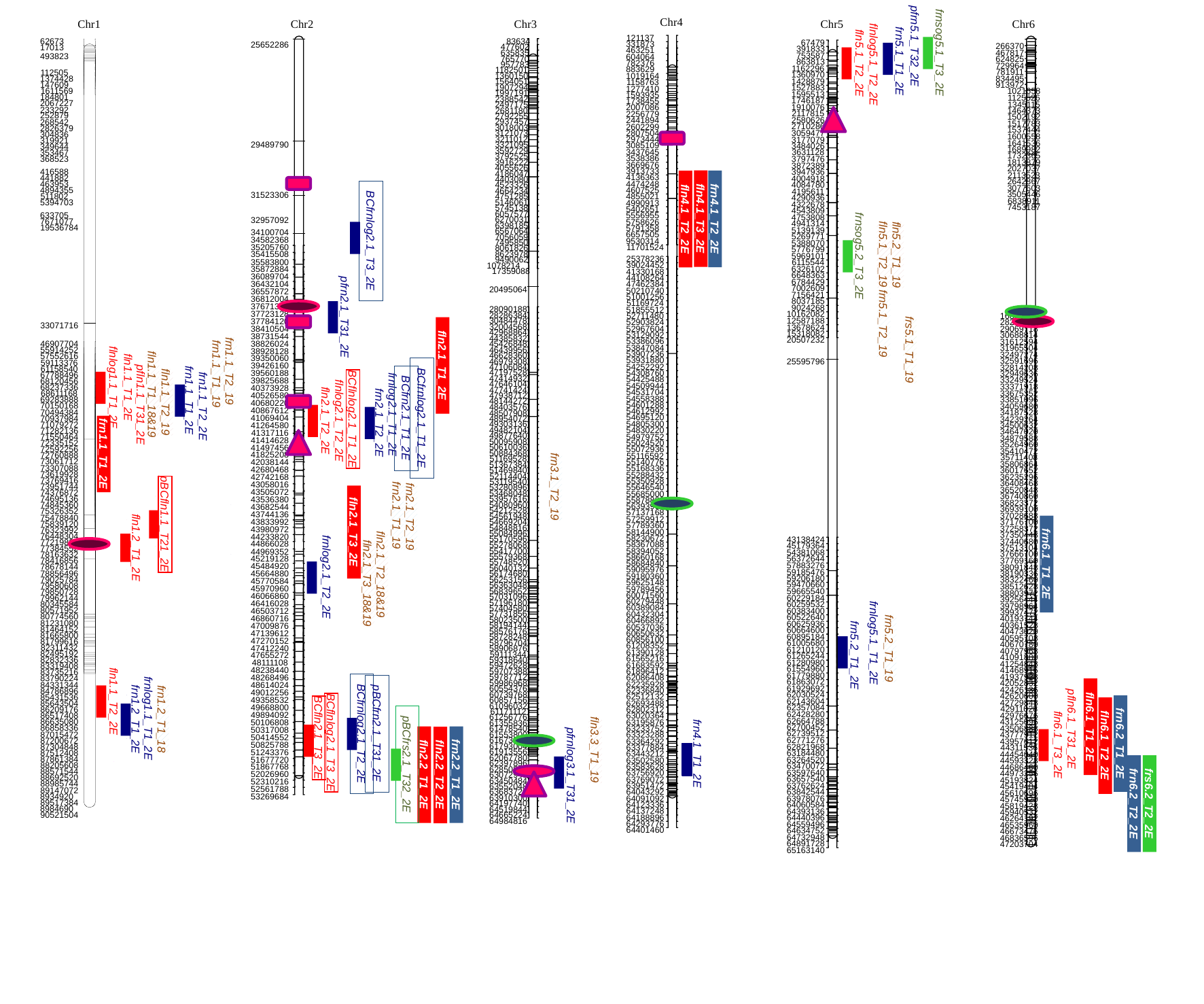

frnsog5.1_T3_2E
Chr5
67479
391833
753587
863813
1162296
1360970
1428879
1527883
1595513
1746187
1910076
2117815
2580626
2710286
3059477
3177079
3484026
3631128
3797476
3872389
3947936
4004918
4084780
4195611
4290936
4322678
4543809
4753808
4941314
5139139
5269771
5388070
5776799
5969101
6115544
6326102
6648363
6784429
7002609
7156421
8037185
9024268
10162082
12587188
13678624
15318082
20507232
25595796
43138424
45179364
54381068
56372644
57883276
59185476
59206180
59470660
59665540
60229184
60259532
60383400
60522640
60625936
60664600
60895184
61005680
61210120
61265244
61280980
61554960
61779880
61863072
61929692
62030524
62143604
62357084
62428280
62664788
62700452
62739512
62771276
62821968
63184480
63264520
63470072
63597640
63657540
63762624
63842544
63978076
64060584
64393136
64440396
64559496
64634752
64732948
64891728
65163140
frn5.1_T1_2E
fln5.1_T2_2E
pfrn5.1_T32_2E
flnlog5.1_T2_2E
frnsog5.2_T3_2E
fln5.2_T1_19
frn5.1_T2_19
frs5.1_T1_19
frn5.2_T1_2E
frnlog5.1_T1_2E
Chr4
121137
331873
463251
604064
782376
883629
1019164
1158763
1277410
1593935
1738455
2007086
2256779
2441894
2602299
2807504
2973444
3085109
3437645
3538386
3669676
3913733
4136363
4474248
4607525
4855021
4990913
5402651
5556955
5758626
5791358
6657505
9530314
11701524
25378236
39024452
41330168
44108264
47462384
50210740
51001256
51169724
51855512
52711480
52903824
52967604
53129092
53386096
53847084
53907236
53931880
54252292
54308760
54425488
54509944
54531704
54558388
54601288
54612992
54695120
54805300
54830220
54979752
55024520
55072936
55116592
55140776
55168336
55288432
55350928
55646540
55685000
55878880
56393908
57137168
57259912
57789360
58144900
58230672
58367088
58394052
58660168
58684840
59095976
59180360
59625148
59789456
60071560
60279448
60389084
60432304
60466892
60537036
60650632
60856100
61208352
61390128
61565216
61683592
61896412
62086408
62235928
62336840
62512132
62693488
62802312
63020364
63195876
63233752
63323288
63364292
63377884
frn4.1_T1_2E
63443212
63502580
63583628
63756920
63769072
63951472
64043292
64091092
64123336
64137248
64188896
64293776
64401460
Chr1
62673
17013
493823
112505
1374428
147609
1611569
184801
2067227
233292
252879
268542
2826379
304836
319921
349644
353467
368523
416588
441882
463953
4894355
511802
5394703
633705
7671077
19536784
33071716
46907704
55914252
57552616
59113376
61158540
67788496
68120456
68237336
68611168
69283888
70150168
70494384
70937984
71079272
71282136
71550464
72335152
72592256
72760888
73061712
73307088
73619928
73769416
73951744
74376872
74695136
74845360
75326352
75478840
75839120
76323992
76448304
77219824
77384552
78163632
78416856
78678144
78856496
79025784
79580608
79850728
79962144
80345584
80571952
80774560
81231080
81464152
81665800
81799616
82311432
82495192
82832336
83319408
83735216
83790224
84331344
84786896
85431536
85643504
86209176
86517408
86635080
86858336
87015472
87200672
87304848
87512408
87861384
88205608
88571544
88692520
88985744
89147072
8934920
89517384
8984690
90521504
flnlog1.1_T1_2E
fln1.1_T1_2E
frn1.1_T1_2E
frn1.1_T2_2E
frn1.1_T1_2E
pBCfln1.1_T21_2E
fln1.2_T1_2E
frn1.2_T1_2E
frnlog1.1_T1_2E
fln1.1_T2_2E
Chr2
25652286
29489790
31523306
32957092
34100704
34582368
35205760
35415508
35583800
35872884
36089704
36432104
36557872
36812004
37671364
37723128
37784120
38410504
38731544
38826024
38928128
39350060
39426160
39560188
39825688
40373928
40526580
40680220
40867612
41069404
41264580
41317116
41414628
41497456
41825208
42038144
42680468
42742168
43058016
43505072
43536380
43682544
43744136
43833992
43980972
44233820
44866028
44969352
45219128
45484920
45664880
45770584
45970960
46066860
46416028
46503712
46860716
47009876
47139612
47270152
47412240
47655272
48111108
48238440
48268496
48614024
49012256
49358532
49668800
49894092
50106808
50317008
50414552
50825788
51243376
51677720
51867768
52026960
52310216
52561788
53269684
BCfrnlog2.1_T3_2E
pfrn2.1_T31_2E
frnlog2.1_T1_2E
frn2.1_T2_2E
BCfrn2.1_T1_2E
BCfrnlog2.1_T1_2E
fln2.2_T1_2E
fln2.1_T1_2E
fln2.1_T2_2E
flnlog2.1_T2_2E
BCflnlog2.1_T1_2E
frnlog2.1_T2_2E
fln2.1_T3_2E
BCfrnlog2.1_T2_2E
BCfln2.1_T3_2E
pBCfrn2.1_T31_2E
BCflnlog2.1_T3_2E
frn2.2_T1_2E
Chr3
83634
477602
635835
765770
957783
1182501
1360150
1564051
1907294
1997191
2388542
2497175
2681180
2792255
2937457
3018003
3121073
3211012
3321095
3592729
3792525
3916222
4055626
4186047
4403080
4523326
4664234
4751285
5146061
5745138
6057577
6270031
6398185
6567064
7056059
7495850
8061826
8623978
9490062
1078214
17359088
20495064
28090188
28286384
30484478
32004568
42968864
44385832
45426848
46439956
46628360
46979308
47106084
47197528
47414932
47646104
47741424
47938712
48144272
48403576
48507908
48954072
49303136
49482104
49877640
50095908
50610036
50884368
51169528
51367384
51469840
52114404
53119540
53280896
53468048
53957616
54080960
54212528
54561948
54669204
54848816
55084996
55170596
55278068
55417700
55579368
55748520
56040132
56174680
56253156
56363048
56839652
57031096
57196180
57404580
57731856
58023500
58194144
58576176
58728248
58796704
58906876
59111344
59318640
59472628
59707388
59787712
59986968
60554376
60739768
60857156
61096032
61171112
61256776
61355836
61478540
61553800
61673804
61793004
61913556
62067780
62397896
62850516
63072948
63450484
63552048
63683720
63910300
64197740
64519844
64665224
64984816
frn3.1_T2_19
fln3.3_T1_19
pfrnlog3.1_T31_2E
Chr6
266370
467817
624825
729964
781911
834495
913972
1021658
1125526
1345115
1464373
1502192
1517783
1537444
1600558
1641536
1689982
1732365
1813879
2027037
2113523
2642867
3077603
3505446
6838911
7453187
16834936
28208920
29069718
30688814
31612594
31965504
32497274
32591696
32814208
32949636
33249524
33371918
33675352
33851096
34059680
34187528
34239264
34500632
34647020
34879588
35264960
35410472
35711408
35806864
36017652
36235296
36408468
36520848
36740860
36823372
36939100
37028688
37176700
37258372
37350448
37440680
37513104
37666700
37769160
38091848
38190336
38322460
38512628
38803972
39256444
39798964
39937472
40193744
40361528
40473020
40595708
40670780
40797968
41091880
41254848
41468916
41937928
42052832
42426136
42620600
42729848
42911028
42976676
43125096
43506888
43777912
43957612
44311536
44454040
44593328
44686460
44973256
45193824
45419404
45610696
45745920
45819428
45940532
46264192
46535960
46673476
46836596
47203704
frn6.1_T1_2E
fln6.1_T3_2E
fln6.1_T1_2E
pfln6.1_T31_2E
frn6.2_T1_2E
fln6.1_T2_2E
frn6.2_T2_2E
frs6.2_T2_2E
fln4.1_T3_2E
frn4.1_T2_2E
fln4.1_T2_2E
fln5.1_T2_19
frn1.1_T2_19
frn1.1_T1_19
fln1.1_T1_18&19
fln1.1_T2_19
pfln1.1_T31_2E
frn2.1_T1_19
frn2.1_T2_19
fln2.1_T2_18&19
fln2.1_T3_18&19
frn5.2_T1_19
pBCfrs2.1_T32_2E
frn1.2_T1_18
fln2.2_T2_2E

## Slide 2
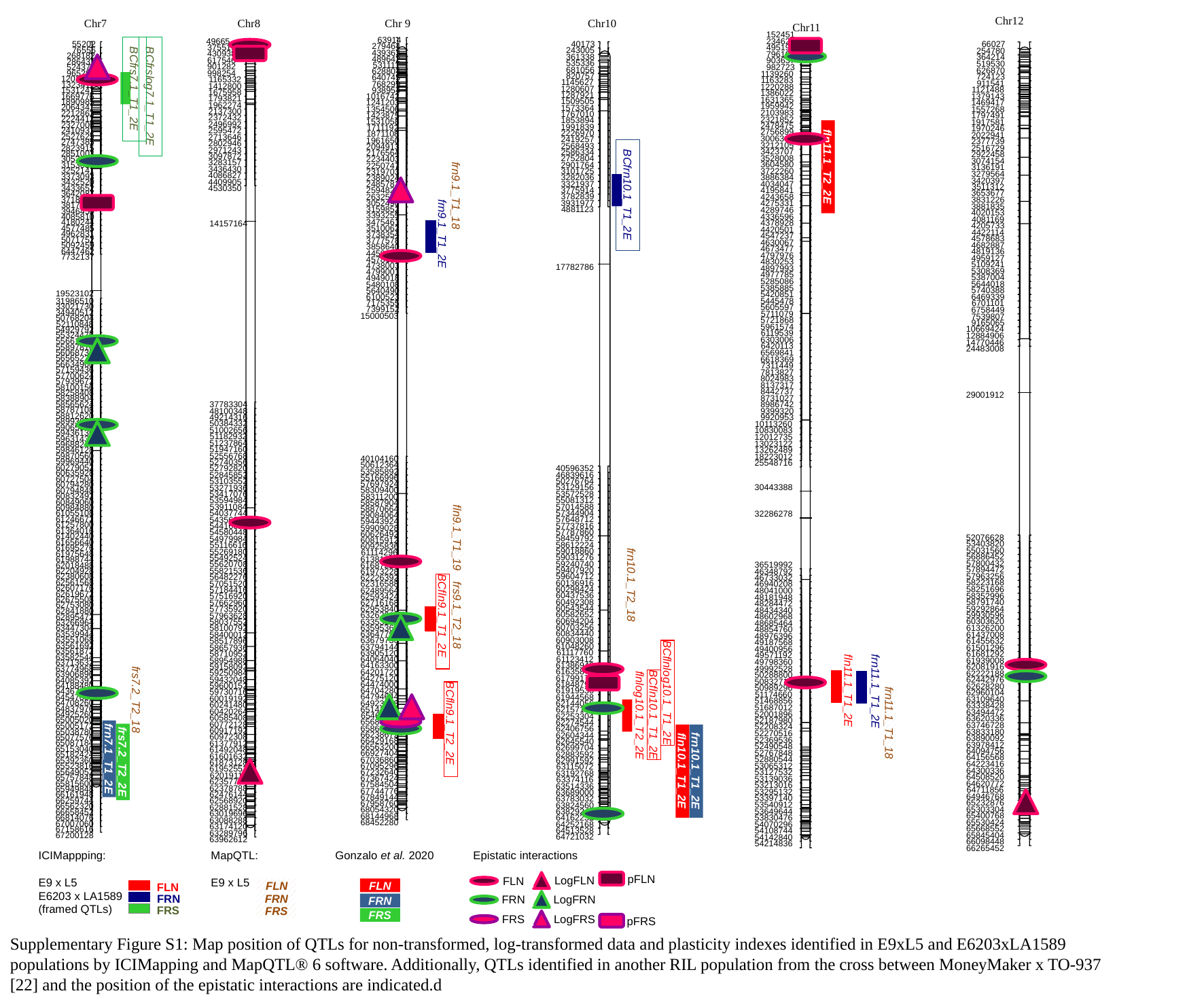

Chr12
66027
254780
364214
519530
626870
724123
911541
1121488
1379143
1469417
1557268
1797491
1917581
1970246
2022941
2377739
2516729
2922458
3074154
3136191
3279564
3420397
3511312
3653677
3831226
3881835
4020153
4081169
4205733
4422114
4578683
4682887
4819136
4959127
5109241
5308369
5387004
5644018
5740388
6469339
6701101
6758449
7539807
9165065
10669424
12884906
14770446
24483008
29001912
52076628
53403820
55031560
56886452
57800432
57894472
57963256
58223168
58251696
58352996
58791740
59292864
59930596
60303620
61326200
61437008
61455632
61501296
61681292
61939008
62081916
62222188
62442976
62628280
62960104
63109640
63338428
63494472
63620336
63746728
63833180
63890092
63978412
64094756
64156568
64223416
64300336
64508520
64620772
64711856
64946768
65232876
65303304
65400768
65530424
65668552
65845404
66098448
66265452
Chr7
55202
76556
268182
286431
524379
965295
1202996
1323411
1531241
1669776
1890985
2064341
2212863
2224412
2327006
2410931
2527629
2747385
2823915
2851003
3053153
3153056
3252141
3373093
3432520
3433652
3642087
3718089
3817239
3946450
4085810
4180244
4577485
4962831
5071752
5092459
6447462
7732137
19523102
31986510
33021730
34940512
50768204
52110848
54929792
55324476
55661008
55897816
56068732
56565224
56634960
57159436
57700624
57939672
58100156
58258408
58388904
58565624
58787108
58812620
58993624
59065048
59436136
59631432
59688240
59846128
59870560
59969440
60279052
60635928
60727504
60794280
60794848
60832492
60849060
60984880
61055108
61246672
61257800
61364016
61402440
61656640
61695276
61975648
61988744
62018488
62204928
62380608
62561568
62607176
62619672
62675508
62753080
62841880
62895472
63266964
63447304
63539944
63551068
63561692
63561872
63582544
63713632
63774968
63906896
64085392
64188480
64367368
64547084
64708260
64837976
64925260
65005020
65005172
65038780
65077576
65087184
65153040
65182424
65392360
65523816
65649052
65757848
65815600
65949848
66161948
66259744
66562320
66658452
66814076
67007060
67158616
67200128
BCfrs7.1_T1_2E
BCfrslog7.1_T1_2E
frn7.1_T1_2E
frs7.2_T2_2E
Chr8
49665
375519
430938
617546
901282
998254
1165332
1412800
1675958
1793821
1962274
2137300
2372432
2496992
2595472
2713646
2802946
2971243
3097872
3283157
3436430
4086827
4409905
4530350
14157164
37783304
48100348
49214316
50384332
51002656
51182932
51237864
51947160
52556768
52740356
52792820
52845852
53103552
53271936
53417076
53594984
53911084
54037744
54356284
54416016
54580448
54979984
55116616
55269180
55492524
55620708
55821536
56482276
57051520
57184416
57516920
57662960
57735920
57963628
58037552
58100792
58400012
58517896
58657936
58710952
58954988
59158092
59250984
59432048
59600152
59730716
60019192
60241480
60420264
60585408
60772128
60917192
60972308
61377912
61492048
61601632
61873128
61952552
62019116
62357772
62378788
62476144
62568920
62881528
63019696
63088288
63174120
63289796
63962612
Chr 9
63914
279464
439363
489642
531111
628804
640745
768299
938954
1016742
1241203
1354506
1423875
1531058
1711192
1871108
1961650
2094913
2176564
2234403
2250747
2319703
2389024
2485781
2594828
2632574
3052490
3159852
3393255
3475461
3510062
3738354
3777578
3858640
4450858
4578159
4738003
4799001
4949018
5480108
5640490
6100523
7175355
7399152
15000503
40104160
50612364
53585892
55166996
57697924
58309400
58311200
58587904
58870664
59084064
59443924
59909028
60626492
60815912
60925836
61114296
61381012
61687220
61973228
62226392
62316588
62489564
62593424
62716168
62953840
63262012
63353208
63595360
63647796
63679736
63794144
63905120
64064040
64163300
64201720
64275120
64474000
64704280
64794644
64923748
65141824
65461128
65686096
65863692
66238672
66429168
66563200
66927404
67036860
67095296
67232640
67367424
67584504
67744776
67849144
67958760
68054320
68144968
68452280
frn9.1_T1_2E
BCfln9.1_T1_2E
BCfln9.1_T2_2E
Chr10
40173
243005
361338
535336
681056
820757
1145627
1280607
1287921
1509505
1573364
1767010
1853894
1991839
2226970
2419257
2568493
2586334
2752804
2901764
3101725
3282036
3321937
3775914
3782839
3931977
4881123
17782786
40596352
46839616
50276764
53129156
53572528
55081312
57014588
57344904
57648712
57737816
57787860
58459792
58612224
59018860
59031276
59240740
59407920
59604712
60136916
60298424
60437536
60492308
60543544
60582652
60694204
60703256
60834440
60903008
61048260
61117760
61123412
61386948
61635872
61799172
61848792
61919536
61944568
62144056
62157104
62253304
62274544
62406756
62604344
62645540
62699704
62883592
62991592
63115072
63192768
63374116
63514336
63689000
63783032
63824560
63829240
64162232
64252168
64513528
64721032
BCfrn10.1_T1_2E
flnlog10.1_T2_2E
BCflnlog10.1_T1_2E
BCfln10.1_T1_2E
fln10.1_T1_2E
frn10.1_T1_2E
Chr11
152451
234626
495194
796108
903637
982723
1139260
1163283
1220288
1386022
1631365
1959942
2103983
2321852
2478475
2756899
3006314
3212105
3423707
3528008
3604580
3722260
3886384
4034047
4195841
4243658
4275331
4289746
4336596
4378928
4420501
4547237
4630067
4673477
4797976
4830253
4897993
4977785
5285086
5385885
5420851
5445478
5605597
5711079
5721868
5961574
6119539
6303006
6420113
6569841
6618369
7311449
7813827
8024983
8137317
8442737
8731027
8986742
9399320
9920953
10113260
10830083
12012735
13023122
13262489
18223012
25548716
30443388
32286278
36519992
46348792
46733032
46940208
48041000
48181948
48284472
48434340
48602968
48685464
48854760
48976396
49187568
49400956
49571192
49798360
49992528
50288800
50832716
50989296
51174660
51468856
51687012
52001896
52187980
52208324
52270516
52369536
52490548
52767848
52880544
53065312
53127532
53139036
53213016
53295132
53397140
53540912
53649644
53830476
54070296
54108744
54142840
54214836
fln11.1_T2_2E
fln11.1_T1_2E
frn11.1_T1_2E
frn9.1_T1_18
fln9.1_T1_19
frn10.1_T2_18
frs9.1_T2_18
frs7.2_T2_18
frn11.1_T1_18
ICIMappping:
E9 x L5
E6203 x LA1589 (framed QTLs)
MapQTL:
E9 x L5
Gonzalo et al. 2020
Epistatic interactions
pFLN
pFRS
LogFLN
LogFRN
LogFRS
FLN
FRN
FRS
FLN
FRN
FRS
FLN
FRN
FRS
FLN
FRN
FRS
Supplementary Figure S1: Map position of QTLs for non-transformed, log-transformed data and plasticity indexes identified in E9xL5 and E6203xLA1589 populations by ICIMapping and MapQTL® 6 software. Additionally, QTLs identified in another RIL population from the cross between MoneyMaker x TO-937 [22] and the position of the epistatic interactions are indicated.d
