# Supplementary material for: Genetic Control of Reproductive Traits under Different Temperature Regimes in Inbred Line Populations Derived from Crosses between S. pimpinellifolium and S. lycopersicum Accessions
Source: Plants (Basel). 2022 Apr 14;11(8):1069. doi: 10.3390/plants11081069 (PMC9027731; doi:10.3390/plants11081069)
Supplement: Supplementary file 1 [file plants-11-01069-s001.zip › Supplementary Figure S2.pptx]

## Slide 1
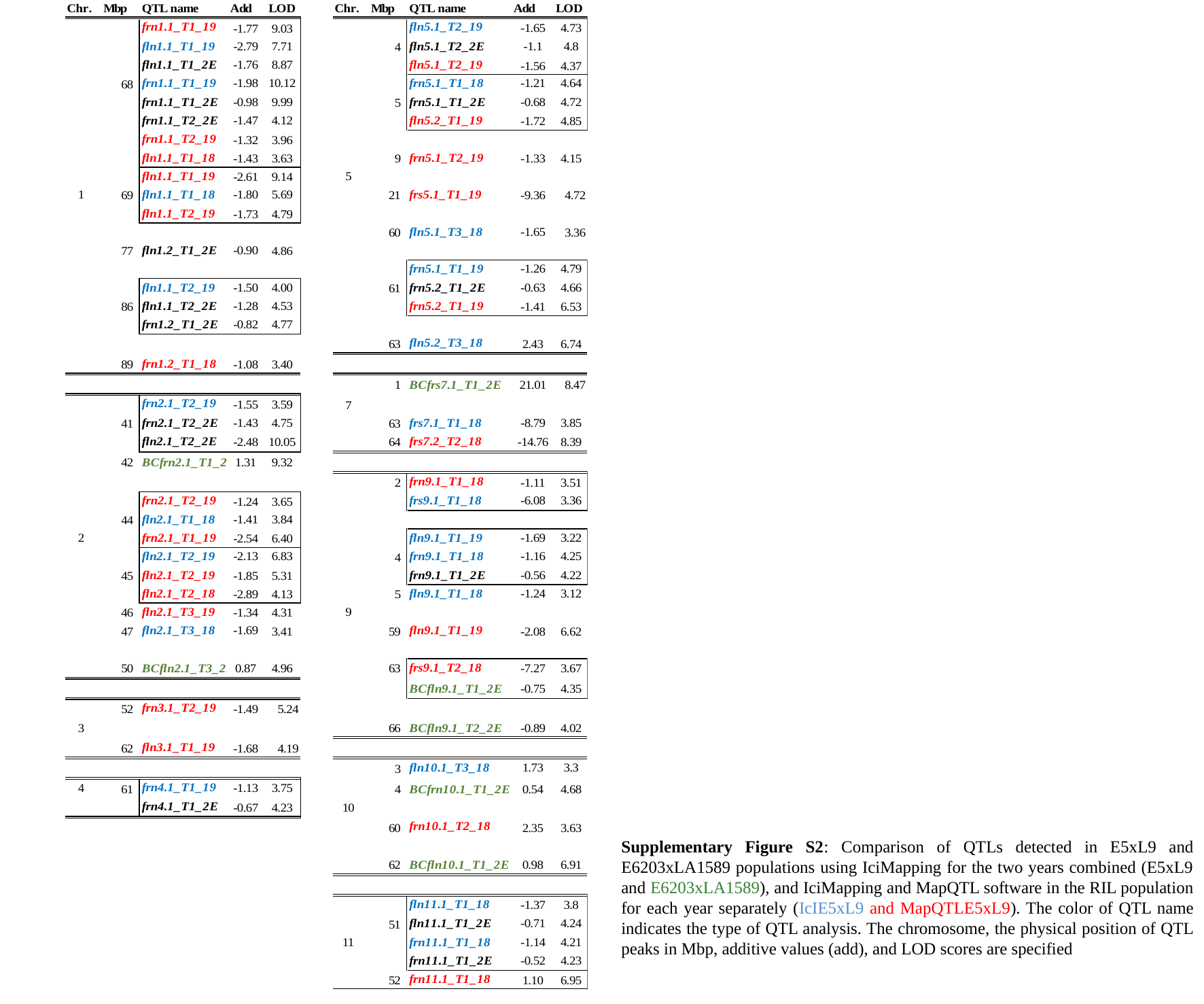

Supplementary Figure S2: Comparison of QTLs detected in E5xL9 and E6203xLA1589 populations using IciMapping for the two years combined (E5xL9 and E6203xLA1589), and IciMapping and MapQTL software in the RIL population for each year separately (IcIE5xL9 and MapQTLE5xL9). The color of QTL name indicates the type of QTL analysis. The chromosome, the physical position of QTL peaks in Mbp, additive values (add), and LOD scores are specified
